# Improved quantitative microbiome profiling for environmental antibiotic resistance surveillance

**DOI:** 10.1186/s40793-021-00391-0

**Published:** 2021-11-18

**Authors:** Amelie Ott, Marcos Quintela-Baluja, Andrew M. Zealand, Greg O’Donnell, Mohd Ridza Mohd Haniffah, David W. Graham

**Affiliations:** 1 School of Engineering, Newcastle University, Cassie Building, Newcastle upon Tyne, NE1 7RU UK; 2 Universiti Teknologi Malaysia, Jalan Iman, 81310 Skudai, Johor Malaysia

**Keywords:** Quantitative microbiome, Hill numbers, Antibiotic resistance, QMRA, River water, Southeast Asia

## Abstract

**Background:**

Understanding environmental microbiomes and antibiotic resistance (AR) is hindered by over reliance on relative abundance data from next-generation sequencing. Relative data limits our ability to quantify changes in microbiomes and resistomes over space and time because sequencing depth is not considered and makes data less suitable for Quantitative Microbial Risk Assessments (QMRA), critical in quantifying environmental AR exposure and transmission risks.

**Results:**

Here we combine quantitative microbiome profiling (QMP; parallelization of amplicon sequencing and 16S rRNA qPCR to estimate cell counts) and absolute resistome profiling (based on high-throughput qPCR) to quantify AR along an anthropogenically impacted river. We show QMP overcomes biases caused by relative taxa abundance data and show the benefits of using unified Hill number diversities to describe environmental microbial communities. Our approach overcomes weaknesses in previous methods and shows Hill numbers are better for QMP in diversity characterisation.

**Conclusions:**

Methods here can be adapted for any microbiome and resistome research question, but especially providing more quantitative data for QMRA and other environmental applications.

**Supplementary Information:**

The online version contains supplementary material available at 10.1186/s40793-021-00391-0.

## Background

Antibiotic resistance (AR) represents a global threat [1]. Between 2014 and 2016, more than one million people died due to drug resistant pathogen infections and increasing death tolls are expected in the future [2]. AR pathogens not only spread through hospitals, but also enter the environment via insufficiently treated sewage [3, 4]. This is especially a problem in emerging countries. Increased economic wealth permits greater access to antibiotics while waste management often lags behind [5]. However, quantifying the extent of environmental AR over space and time is difficult because methods are not standardized, with researchers using different measures of AR (e.g. antibiotics, antibiotic resistant genes, ARGs; antibiotic resistant bacteria, ARBs; and mobile genetic elements, MGEs) across studies [6]. Ideally, bacterial hosts of ARGs should be tracked [7], but reliable molecular methods that couple bacteria species and ARG abundances (e.g. epicPCR [8], Hi-C [9]) are still in their infancy. Further, linking microbiome characteristics from DNA sequencing with quantitative ARG data is an unfulfilled aspiration for studying environmental AR [10, 11]. This restricts our ability to perform realistic Quantitative Microbial Risk Assessments (QMRA) needed to quantify true risks of environment AR exposures [12, 13]. Correlation-based methods can develop hypotheses to guide future experimental work but they are restricted due to technical biases introduced from DNA sequencing [7, 14, 15].

**Fig. 1:**
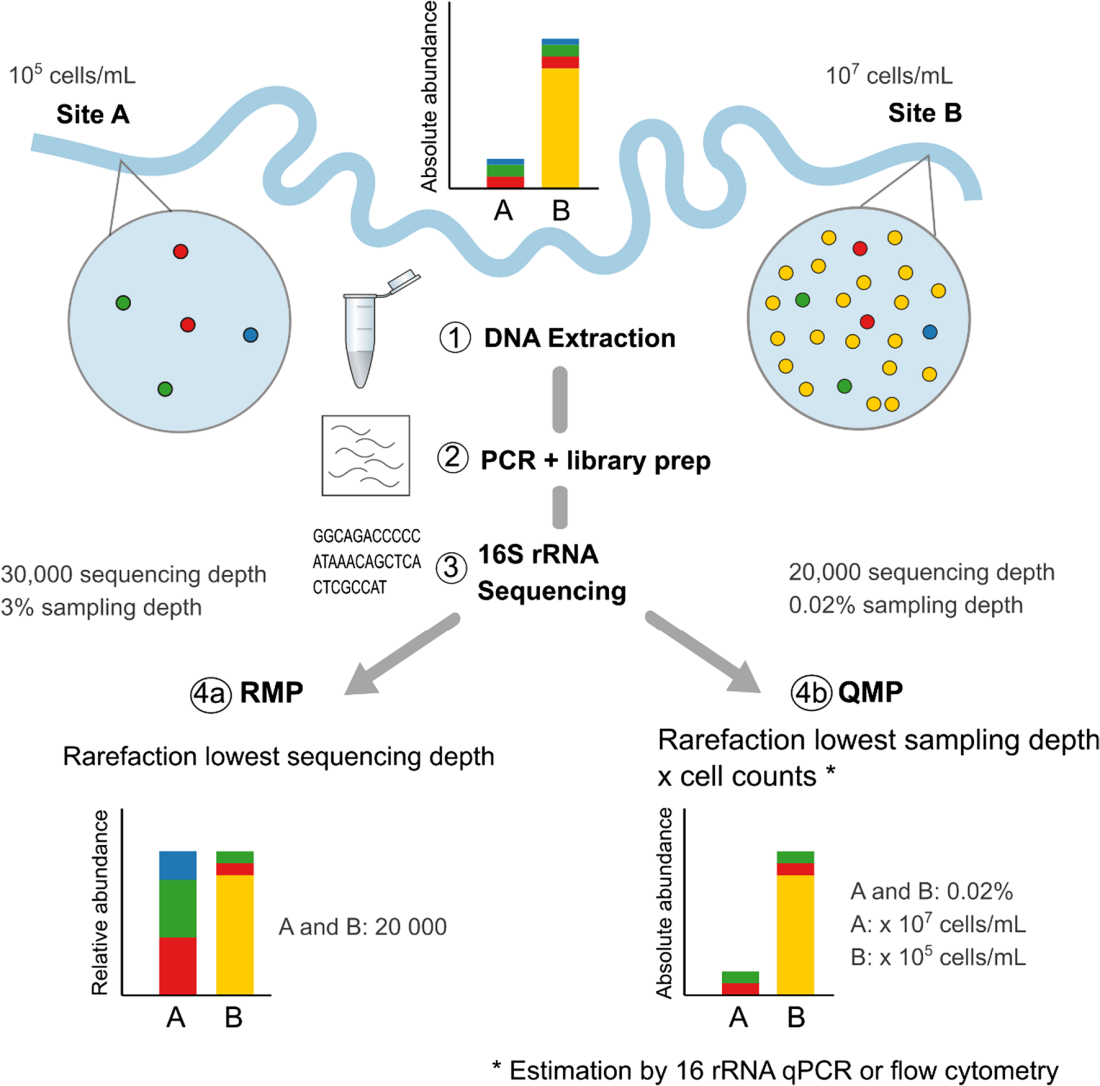
Schematic explaining relative (RMP) and quantitative (QMP) environmental microbiome profiling. Both, the RMP and QMP approach do not correct for biases introduced by sample collection, DNA extraction, PCR or library preparation. QMP approach based on [17]. While cell counts vary 100-fold between river water samples A and B, sequencing depth (= reads) per sample is independent of cell counts in next-generation sequencing. The RMP approach rarefies to lowest sequencing depth per sample, calculating relative abundance (%), which results in sample A being sequenced more intensively than sample B. The relative abundance profile poorly reflects the real environmental taxa distribution. The QMP approach corrects for sampling intensity by rarefying to the lowest sampling depth (= sequencing depth divided by cell counts) and then multiplies the rarefied taxon abundance with estimated cell counts to obtain absolute abundances (here per mL river water). As the blue taxon was equally abundant in A and B, the fact that it is included for RMP sample A can be considered an artefact of uneven sampling intensity

Next-generation sequencing (NGS) data are inherently compositional, providing relative abundance information at best [16]. It is impossible to measure absolute growths or declines of particular microorganisms solely with relative abundances as, for example, the increase of one taxon leads to the concurrent decrease of other(s) [17] (Fig. 1). Analysing relative abundance data using inappropriate statistical tools (such as parametric statistical tests e.g. ANOVA and measures of correlation e.g. Spearman's rank correlation) can yield up to 100% false detection rates and their application contributes to a general lack of reproducibility among microbiome studies [18, 19].

While compositional approaches are available [16], the gold standard requires cell count estimates to calculate absolute abundances [7, 19]. Such a quantitative approach can also correct sequencing data for sampling intensity to account for varied microbial loads across samples [17] (Fig. 1). Despite environmental studies routinely providing cell count estimates, these data are rarely used to calculate absolute microbial taxon abundances [20], with no studies correcting for sampling intensity [17]. Instead of relative microbiome profiling (RMP), we contend that environmental researchers should use quantitative microbiome profiling (QMP, [17]) to represent a more accurate picture of relationships between microbiomes, resistomes and metadata, guiding future QMRA applications.

The RMP approach rarefies to the lowest sequencing depth per sample, calculating relative abundance (%). In contrast, the QMP approach as introduced by [17] corrects for sampling intensity by rarefying to the lowest sampling depth (= sequencing depth divided by cell counts) and then multiplies the rarefied taxon abundance with estimated cell counts to obtain absolute abundances (e.g. per mL surface water, Fig. 1).

**Fig. 2:**
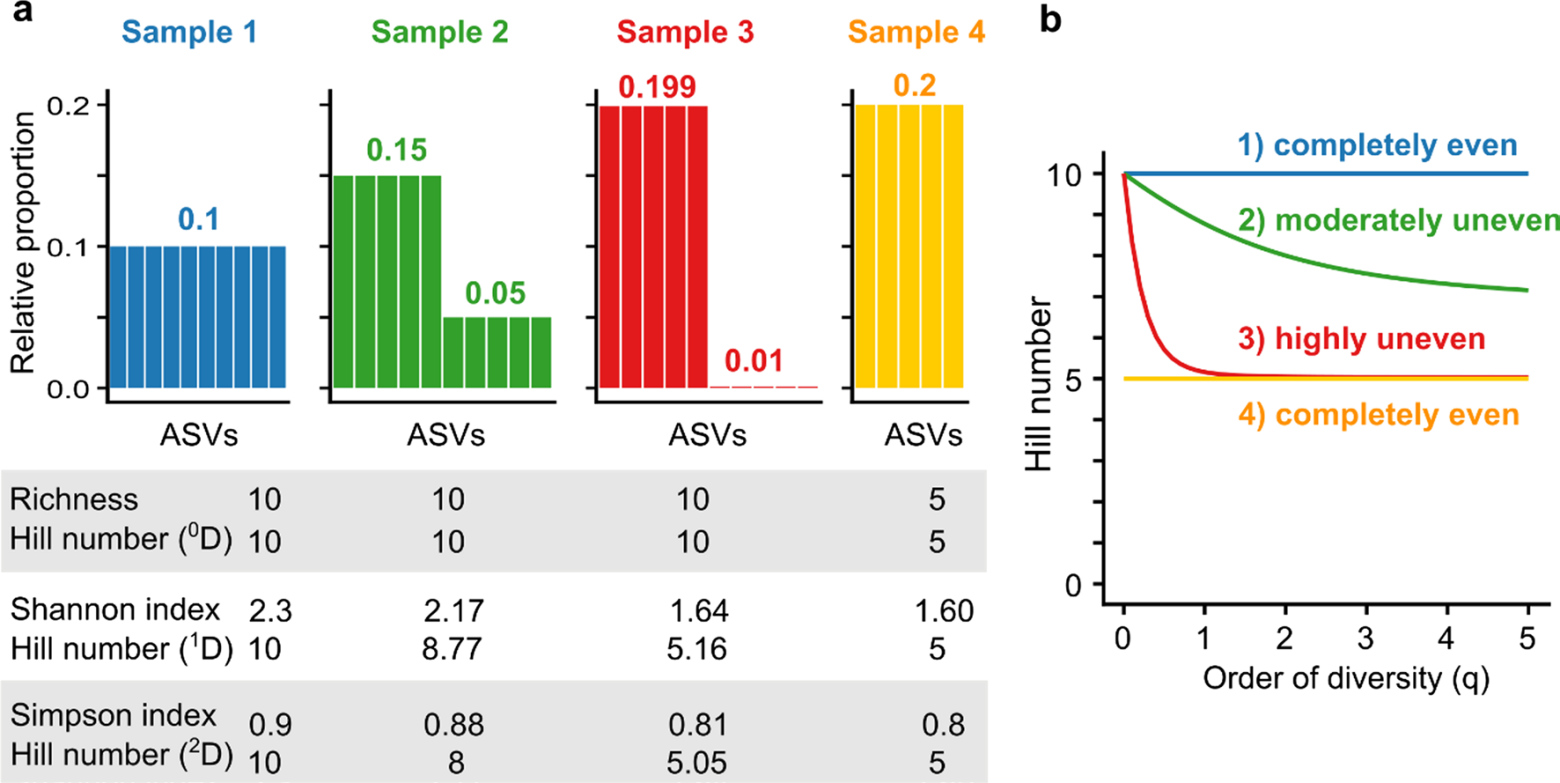
Schematic explaining the relationship between microbiome composition, diversity indices (richness, Shannon index and Simpson index), Hill numbers ^q^D (**a**) and diversity profiles for four theoretical systems (**b**). Figure adapted from [30]. For sample 1 and sample 4, all amplicon sequence variants (ASVs) are evenly distributed, so Hill numbers of all orders of diversity (q) stay the same within sample 1 and sample 4. As sample 4 has half the amount of equally abundant ASVs to sample 1, Hill numbers also half, in contrast to the Shannon index or Simpson index. At q = 0, only richness is considered, ignoring relative abundance. Consequently, for q = 0, Hill numbers for samples 1, 2 and 3 are the same. For q > 0, Hill numbers decrease as the importance attributed to abundant ASVs increases. As sample 3 is dominated by 5 ASVs, Hill numbers ^1^D and ^2^D approximate 5. The diversity profile (**b**) shows the number of ASVs and evenness of the four theoretical systems. A flat profile indicates evenness

Characterising and comparing anthropogenic impacts on environmental microbiomes (e.g. sewage entering rivers, waste leaching, land runoff etc.) is generally hindered by the use of varying microbial diversity indices across studies [21–23]. For a more meaningful quantification, 'diversity' needs to be unambiguously defined and applied in microbiome research [24]. Common diversity indices such as the Shannon and Simpson index do not measure diversity, but uncertainty and probability, respectively [23]. In contrast, Hill numbers (Fig. 2) provide a statistical framework that unifies and generalizes popular indices, and are intuitive and flexible enough to address a wide range of scientific questions [23, 25, 26]. Hill numbers were first proposed almost 50 years ago [26], but despite their continued appraisal [23–25], their use in microbiome research is rare [27, 28], especially for environmental microbiomes [29]. Hill numbers (^q^D, where superscript q describes the order of diversity) also have several additional advantages over other common diversity indices (Table 1).

**Table 1:** Advantages of Hill numbers in comparison to standard diversity indices

1	Interpretation of the measure and its measurement unit is always the same in 'effective numbers of species', i.e. the number of equally abundant species (or for DNA based approaches operational taxonomic unit (OTU)/amplicon sequence variant (ASV) [30]) required to generate an identical diversity [26]
2	Hill numbers double as the amount of equally common species doubles (called the 'doubling principle'), which allows more meaningful calculations of statistical significant changes [23]
3	The sensitivity towards abundant and rare species can be modulated with a single parameter with Hill numbers (order of diversity – q)
4	Hill numbers can be computed taking into account phylogenetic or functional relationships among species (e.g. similar to Faith’s Phylogenetic Diversity [30])
5	Hill numbers were originally developed for abundance data, but can also be applied to incidence data [25]
6	Within the Hill framework, the diversity of a system can be partitioned, so α-diversity (average diversity of subsystems) multiplied by β-diversity (difference between subsystems) gives γ-diversity (entire diversity of the system) [31, 32]
7	Multiple (dis)similarity measurements derived from β-diversities can be calculated from Hill numbers with some being equal to other popular indices e.g. Unifrac [25]
8	The calculation of Hill numbers is straight-forward and can easily be implemented into existing bioinformatic pipelines [33]

Despite clear advantages in using Hill numbers [30] and the QMP approach [17] for improving reliability and comparability of environmental microbiomes, their application is rare [17, 27], and to our knowledge, has never been combined. Here we provide a workflow for combining QMP (based on parallelization of amplicon sequencing and 16S rRNA qPCR data to estimate cell counts) with absolute resistome profiling (based on high-throughput qPCR for almost 300 ARGs and MGEs) to monitor AR in an impacted river. Such absolute microbiome profiling bypasses compositional effects in the reconstruction of microbiota interaction networks, allowing one to investigate correlations of taxa with ARGs and MGEs essential for QMRA. We also show the benefits of using the unified Hill number diversity framework to compare microbial community dynamics over space and time and confirm how misleading RMP approaches are for interpreting environmental microbiome and resistome data.

## Methods

### Sample collection and DNA extraction

We collected river water samples (3 technical replicates x 1 L) from the Skudai catchment, Malaysia (288 km^2^, Additional file 1: Fig. S1) at eight sampling points (6 × main river and 2 × tributaries) during five sampling trips to capture seasonality (1 × November 2017, 2 × March 2018 and 2 × July 2018). The catchment is located in a humid tropical climate and is characterised to equal parts by agriculture (of that, 80% oil palm, 20% rubber plantations), forest and built-up areas [34, 35]. In total, 38 samples (with each three technical replicates) were collected with five biological replicates for the main Skudai river (S1, S2, S5, S6, S7, S8) and four biological replicates for the tributaries Melana (M5) and Senai (Se1). Some data related to this analysis was included in a previous manuscript proposing surrogate markers for predicting AR 'hot spots' in rivers where limited data are available (e.g., physico-chemical and ARG data from 30 samples [36]). Here we focus on new methods of data analysis using some of the same data but used in a different manner.

On-site, we monitored river water temperature and dissolved oxygen contents. Between 80–250 mL of river water was filtered onto 0.22 μm cellulose-nitrate filters to extract DNA with the FastDNA SPIN kit for soil (MP Biomedicals). DNA was cleaned with the QIAquick Nucleotide Removal Kit (Qiagen). DNA quality and quantity were measured with NanoDrop and the Qubit dsDNA HS assay (both Thermo Fisher Scientific), respectively. The three technical replicates were pooled to have sufficient DNA for downstream processes. DNA was stored at − 20 °C.

### 16S rRNA qPCR to estimate cell concentration

16S rRNA qPCR assays were performed in triplicate with 16S rRNA 1055f-1392r primers [37] and SsoAdvanced Universal SYBR Green Supermix (Bio-Rad) on the BioRad CFX C1000 System (Bio-Rad) following thermocycle program: (i) 2 min of initial denaturation at 98 °C, and 40 cycles of (ii) 5 s denaturation and 98 °C, and (iii) 5 s annealing/extension at 60 °C [38]. Melt curve analysis and gels were performed. DNA samples were diluted to a working solution of 5 ng/µL and an internal control DNA (gfp_qPCR_f: TCGGTTATGGTGTTCAATGC; gfp_qPCR_R: GACTTCAGCACGTGTCTTGTAG) was used as inhibition controls for the qPCR. Standard curves of each set of primers were constructed using plasmid clones of the target sequences of between 10^2^ and 10^8^ copy numbers, used in parallel with each qPCR run. Cell concentration was estimated by dividing the 16S rRNA concentration by 4.1, the estimated average 16S rRNA GCN per bacterium [39]. We did not incorporate individual 16S GCN adjustments on the sequencing reads [17, 40] as current correction approaches were found to introduce rather than reduce biases [41]. The resolution of Illumina MiSeq often only allows ASV characterisation to genus level, but already within species, 16S rRNA gene copy number (16S GCN) can vary widely (e.g. 6 to 11 16S GCN for *Escherichia coli* [42]).

### High-throughput qPCR to quantify the resistome

High-throughput qPCR (HT-qPCR) of ARGs and MGEs was performed using SmartChip Real-Time PCR (Wafergen). A total of 296 primer sets (Additional file 1: Table S8) were used to detect 283 ARGs (52 β-lactams, 51 non-specific efflux pumps, 46 MLSBs, 39 tetracyclines, 36 aminoglycosides, 32 vancomycins, 11 others, 9 FCA, 7 sulfonamides), 12 MGEs (8 transposases, 4 integrases) and one 16S rRNA gene as previously described [43, 44]. Amplification efficiency had to be within the range of 90%-110% and was only confirmed when all three technical replicates were positive. Relative copy number of ARGs and MGEs were calculated and transformed to absolute copy numbers by multiplying with 16S rRNA concentration for each sample. ARG and MGE cell concentrations were estimated by dividing the 16S rRNA concentration by 4.1, the estimated average 16S rRNA GCN per bacterium [39].

### 16S rRNA sequencing and bioinformatics

The hypervariable V4 region 515F-806R [45] of the 16S rRNA gene was sequenced on the Illumina MiSeq platform with V2 500 cycle chemistry at NU-OMICS, Northumbria University, UK. Sample preparation and sequencing followed the Schloss MiSeq Wet Lab SOP [46] with the only deviation of spiking a 4.5 pM library, as opposed to 4 pM. Sequencing included a positive control (mock community, ZymoBIOMICS Microbial Community DNA Standard, Zymo Research), negative control (water), and extraction control (extracted water). Raw sequences were processed with QIIME2 v.2019.4 [47]. Reads were denoised into ASVs with DADA2 [48, 49], assigning ASVs to genus level with the SILVA reference database (v 138) [50–52]. The V4 primer region 515F-806R was extracted from the SILVA 138 SSU NR99 dataset to retain more sequences within this region as opposed to using primer sequence to find and remove the corresponding region in the QIIME2 environment [53]. The SILVA 138 V4 classifier was trained with the machine learning software library scikit-learn v.0.20.0 using Naïve Bayes methods (*fit-classifier-naive-bayes* [54]) through the *feature-classifier* plugin [55]. The taxonomy was assigned through the same plugin, using the sklearn-based taxonomy classifier (*classify-sklearn* [54]). Accounting for MiSeq bleed-through between runs [56], rare ASVs of less than 0.1% of the mean sample depth were removed. The taxonomy and ASV table biom file [49] were produced for downstream analysis in R [57] with the phyloseq (v 1.34.0) [58] and vegan (v 2.5–7) [59] package. ASVs not classified at phylum level were removed, resulting in a total of 2735 taxa for 38 samples with minimum 12,712 and maximum 83,570 reads.

### Quantitative and relative microbiome profiling

For QMP, we rarefied samples to an equal sampling depth (ratio between sequencing depth and cell counts (Additional file 1: Fig. S2)) with the R function *rarefy_even_sampling_depth* (seed 711) [17]. Reads were not corrected for individual 16S rRNA GCN. The resulting rarefied abundances were multiplied with the estimated cell concentration per sample to obtain absolute microbial taxa abundance per mL of river water. For RMP, we rarefied sampled to an equal sequencing depth of 12,712 (seed 711), resulting in relative microbial abundances.

### Rank-based RMP and QMP comparisons

We analysed ASV rank order changes between the RMP and QMP approach with the rank-biased overlap (RBO) measure and a genus co-occurrence network based on Spearman’s correlation. RBO is a similarity measure on ranked lists, developed to measure the expected overlap of indefinite rankings [60]. RBO does not require every item to appear in both rankings, is not tied to a particular prefix length and its top-weightedness can be adjusted. For the latter, parameter *p* determines the strength of the weighting to top ranks. Raising *p* increases the depth of comparison, e.g. for *p* = 0.9, *p* = 0.95 or *p* = 0.97, 85% of the RBO measure focus on the first ten, first 20 or first 50 results, respectively [60]. We calculated RBO on the most abundant 100 ASVs with *p* = 0.95 to top-weigh the first 20 results in R with the package gespeR (v 1.23.0) [61].

For the co-occurrence patterns, we first removed unclassified or ambiguously defined ASVs at genus level and then selected ASVs present in at least 85% of samples based on the QMP data (= 24 ASVs). The same 24 ASVs were also selected in the RMP data. We defined and visualised taxon-taxon associations by Spearman’s correlations between pairs of taxa with Benjamini–Hochberg multiple testing correction in R with the packages psych (v 2.1.3) [62] and corrplot (v 0.84) [63].

### Resistome volcano plot

We assessed the difference in log_10_ ARG and MGE river water concentrations between up- and downstream (S1 to S8) with the Welch’s t-test, applying Benjamini–Hochberg P adjustment to correct for multiple testing. We plotted the log_10_ fold change against statistical significance in a volcano plot with the R package EnhancedVolcano (v 1.8.0) [64].

### Network analysis for microbiome and resistome correlations

We investigated microbiome and resistome co-occurrence by calculating all possible pairwise Spearman’s rank correlations among bacterial orders, ARGs and MGEs present in the river water samples (n = 38). Only statistically robust correlations with Spearman’s ρ > 0.8 and Benjamini–Hochberg multiple testing corrected *P* < 0.01 [65] were included in the network. Network analysis was performed in R with visualisation including topological property calculations in Gephi (v 0.9.2) [66].

### Hill diversity analysis

Abundance-based Hill numbers and diversity profiles for RMP and QMP were calculated and plotted with the hilldiv R package (v 1.5.1) [33]. The Sørensen‐type overlap dissimilarity measure for q = 1 was used to quantify the effective average proportion of nonshared ASVs in the catchment and visualised in a NMDS plot. As the Hill number ^q^D equation [23, 30] is not defined for q = 1, the R package hilldiv calculated ^q^D for this case with q = 0.99999 (Eq. 1).

1 $${}_{{}}^{q} D = \left( {\mathop \sum \limits_{i = 1}^{S} p_{i}^{q} } \right)^{{1/\left( {1 - q} \right)}}$$

^q^D Hill number, q Order of diversity, S Species richness, p_i_ Proportional abundance of species i.

### Statistical analysis and graphics

We performed all statistical analysis in R (v 4.0.5) [57]. We composed graphics using ggplot2 (v 3.3.3) [67] with finalisations in Inkscape (v 1.0.2) [68] except for where stated differently. The Skudai catchment map was composed in ArcGIS (v 10.6.1) [36, 69]. To assess statistically significant difference in microbiomes and resistomes between upstream (S1) and downstream (S8), we tested for normality with the Shapiro–Wilk test, followed by comparisons with the Welch’s-test [70]. Effect size was measured with Cohen’s D with the R package effsize (v 0.8.1) [71].

## Results

### Relative and absolute microbial taxa abundances

For this study, we collected river water samples in a Malaysian rural-to-urban catchment from eight sampling points over five field trips in different seasons (total n = 38 with four to five biological replicates per site, see Additional file 1: Fig. S1). Our previous sub-study for this catchment found no large statistically significant seasonal effects for water quality and resistome data [36]. Consequently, mean concentrations with standard deviations are reported per sampling point across seasons. We estimated river water cell concentrations with 16S rRNA qPCR, correcting for multiple 16S rRNA gene copies per cell. In the catchment, cell counts varied more than 100-fold across samples with mean upstream concentrations of (9 ± 3) x 10^5^ cells/mL (S1) and mean downstream concentrations of (2 ± 1) x 10^7^ cells/mL (S8) (Additional file 1: Fig. S2).

River water microbiomes were assessed by 16S rRNA sequencing with Illumina MiSeq, classifying ASVs to genus level. After data quality filtering, reads varied from 12,712 to 83,570 (median 28,187, Additional file 1: Fig. S2). Sampling depth (i.e., reads/cell count) was highest in upstream samples (S1; mean 3.4%), with lower sampling depths obtained elsewhere in the catchment (mean 0.16–0.59%, Additional file 1: Fig. S2). The lower cell counts upstream resulted in S1 samples being 21 × more intensely sampled in the microbiome analysis than the most downstream site, S8 (Additional file 1: Fig. S2).

**Fig. 3:**
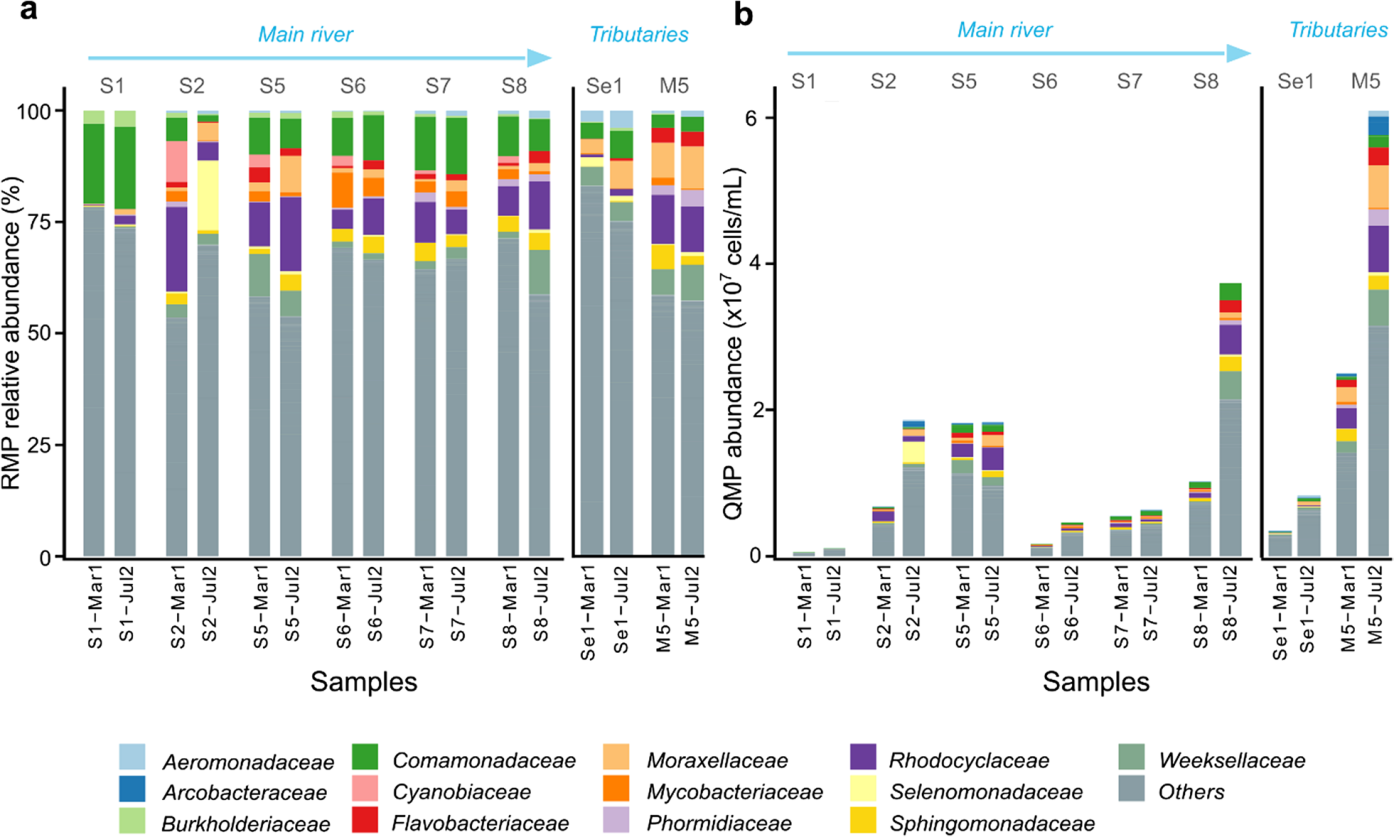
Barplots showing the 20 most abundant ASVs grouped into families with remain pooled into 'Other' for the relative (RMP; **a**) and quantitative (QMP; **b**) microbiome profiling approach, analysing river water samples from eight sampling points for two sampling campaigns (March and July 2018, n = 16)). See Additional file 1: Fig. S3 for all 38 samples

For RMP normalization, samples were rarefied to equal sequencing depth (i.e., number of reads per sample; here 12,712 reads, Additional file 1: Fig. S4). Despite known problems [72], the RMP approach remains the common practice in environmental microbiome research to calculate relative abundances of taxa (Fig. 3) [73]. For QMP [17], samples were rarefied to equal sampling depth (here 0.05%) and multiplied with the estimated cell counts per sample to obtain absolute abundance of taxa per mL river water (Fig. 3). In contrast to [17], individual 16S rRNA gene copy number (16S GCN) adjustment was not performed because related methods are imprecise, introducing additional bias [41].

The most abundant ASVs (based on QMP, Additional file 1: Table S1) were *Cloacibacterium, Acinetobacter, C39* (genus level), and *Comamonadaceae* (family level). When comparing taxa changes across the catchment, the RMP barplot (Fig. 3a) might lead an inexperienced researcher to misleading conclusions. For example, the RMP barplot might be read as *Comamonadaceae* decreasing as one moves downstream (S1 → S8). However, when one takes cell counts into consideration (Fig. 3b), *Comamonadaceae* concentrations actually increase from up- to downstream, which appears logical given progressive waste inputs along the river.

As relationships between microbiomes and metadata are often explored using non-parametric rank-based methods, we assessed whether the ASV rank order was conserved in the QMP vs RMP approaches. Out of the 20 most abundant ASVs determined with QMP, 16 also were present in the top 20 ASVs from the RMP approach, but only three ASVs were at the same rank order in both listings (Additional file 1: Table S1). Assessing the similarity of the rank order of the 100 most abundant ASVs with the rank-biased overlap for top-weightedness [60], we found that only 32% of the QMP and RMP results were in common (score 0.32 with *p* = 95, focussing 86% of the weight on top 20 ASVs), suggesting the two methods providing different pictures of the system—RMP only provides composition, whereas QMP provides composition and abundance in tandem.

**Fig. 4:**
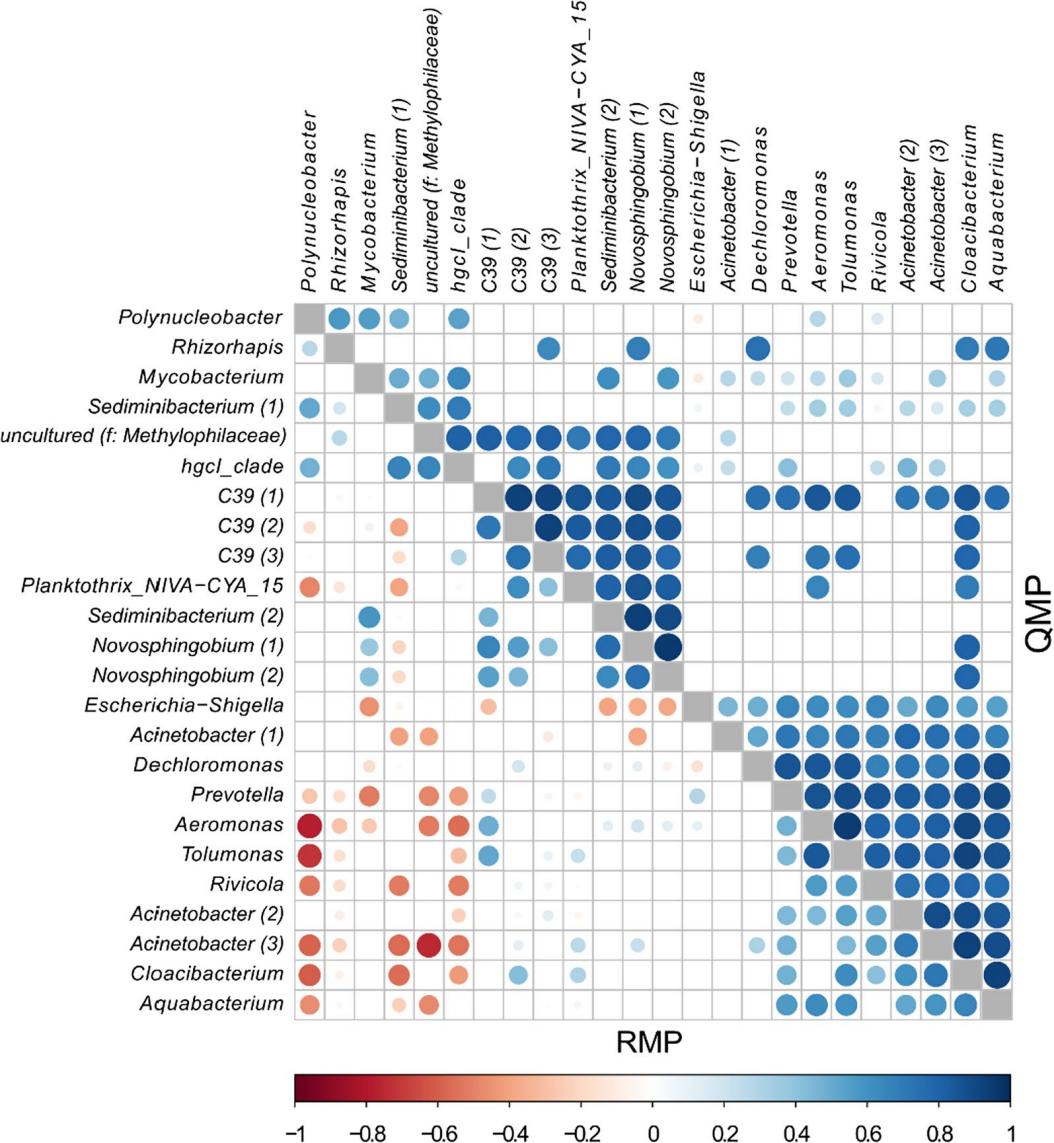
Co-occurrence patterns for ASVs detected in at least 85% of the samples based on relative (RMP) and quantitative (QMP) microbiome profiling. ASVs are labeled based on their genus name. Where different ASVs have the same genus name, numbers in parentheses differentiate those. Pairwise correlations between taxon abundances were calculated, and significant correlations (Benjamini–Hochberg adjusted test, *P* < 0.05) are represented by circles, the colour and size of each circle represent the correlation coefficient (Spearman’s ρ). f: family

Correlation analyses are often used to infer taxon-taxon interactions [14]. Constructing RMP and QMP genus co-occurrence networks (Fig. 4), we detected a much larger number of significant co-varying genus pairs in the QMP than RMP network (249 versus 116). The RMP network also was dominated by negative correlations. None of the moderate to strong RMP correlations (*P* < 0.05, Spearman’s ρ − 0.5 to − 1) were detected in the QMP correlation matrix (Fig. 4).

### Hill numbers for microbial diversity

**Fig. 5:**
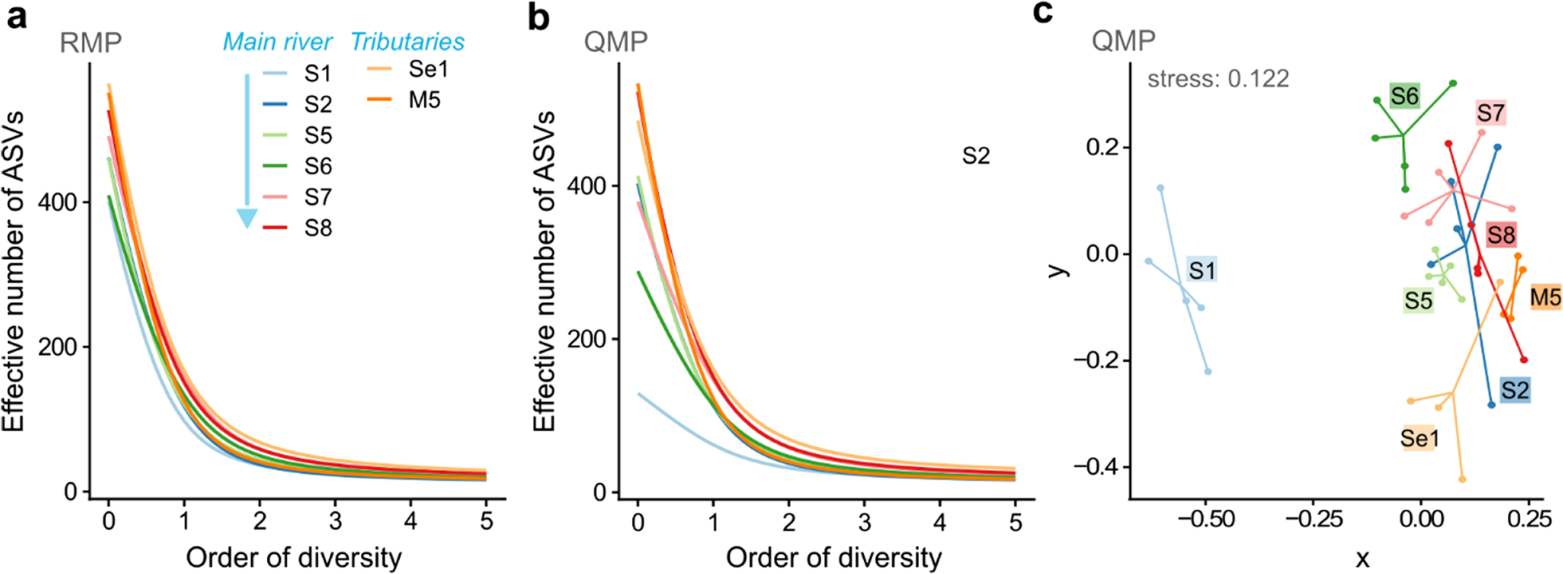
Microbial diversity calculated within the Hill framework across the river catchment. Hill diversity plots represent α-diversities per sampling point based on the relative (**a**) and quantitative (**b**) microbiome profiling approach for varying q values. NMDS Sørensen‐type overlap dissimilarity plot (**c**) is based on β-diversity, calculated for the QMP data with q = 1. Data represented (n = 38) for the eight sampling points is based on five biological replicates for the main river (S1, S2, S5, S6, S7, S8) and on four biological replicates for the tributaries (Se1, M5)

Within the Hill framework, microbial diversity can be calculated for subsystems (α-diversity; the sampling locations), the entire system (γ-diversity; the river catchment), and the difference between subsystems (β-diversity; between sampling points), all expressed using one unit, the effective number of ASVs [30]. The importance of 'richness' (ASV count in a community) and 'evenness' (equality of ASV frequency in a community) to the overall diversity can be modulated with the parameter q [74]. For diversity of order zero (q = 0), the Hill number is a ‘richness’ value because it becomes insensitive to ASV frequency, which overweighs rare ASVs. At q = 1 (exponential of Shannon index), ASVs are weighed by their frequency without favouring rare or abundant ASVs. For q = 2 (inverse of Simpson index), abundant ASVs are overweighted [23]. While specific q values can be selected to calculate diversity, using α-diversities at q = 0, q = 1 and q = 2 together allows one to assess the degree of dominance in a community (Additional file 1: Fig. S5). This information can be summarized in a 'diversity profile', a graph of diversity versus q, visualising the contributions of richness and evenness to a community’s diversity (Fig. 5). The richer a community (higher ASV count), the higher the graph starts, whereas the more uneven the community (few dominant ASVs), the steeper the slope of the graph [23].

Microbial diversities at each sampling point in the RMP diversity profile were closely aligned, with clearer differentiation seen for the QMP data (Fig. 5a, b). Both approaches showed microbial diversity was lower upstream (S1) than elsewhere in the catchment, but spatial differences were smaller using RMP (Additional file 1: Fig. S5). This trend also was observed when calculating the Shannon and Simpson index (Additional file 1: Fig. S4). Further, γ-diversity of the catchment was higher using the RMP versus the QMP approach, but the values for two approaches converged for q > 0. For RMP, γ-diversity in effective numbers of ASVs was 2721 (q = 0), 338 (q = 1) and 96 (q = 2) and for QMP, the values were 2428 (q = 0), 328 (q = 1) and 96 (q = 2).

Results from the RMP and QMP approach differed most in their diversity calculations for the least impacted upstream sampling point S1 (mean difference α at q = 0 was 272 effective number of ASVs, Additional file 1: Fig. S5) with the QMP approach better correcting for varying sampling depths (Additional file 1: Fig. S2), thus avoiding 'over-sequencing'. For the QMP approach (Fig. 5b), the upstream microbial community (S1) was significantly less diverse for q = 0 and q = 1 than the farthest downstream (S8) (Welch’s t-test with P < 0.05 and large Cohen’s D effect size < -0.8, Additional file 1: Table S2). At S1, the microbial community also was more even than at any other sampling point downstream (Fig. 5b).

Comparing the α-diversities for the tributaries Se1 and M5 (Additional file 1: Fig. S5) further shows the benefit of reporting Hill numbers at varying q values. While the tributaries have similar diversities at q = 0 (richness), the diversities for q > 0 (taking frequency into account) decrease more rapidly for the heavily polluted M5 [36], showing a more uneven microbial community in comparison to the less polluted Se1 (Fig. 5b, Additional file 1: Fig. S5).

Within the Hill framework, dissimilarity matrices are based on β-diversities [31, 32]. We used the Sørensen‐type overlap dissimilarity measure for q = 1 to quantify the effective average proportion of nonshared ASVs in the catchment [33] (Fig. 5c). The NMDS plot shows the changing community structure as one moves from rural upstream (S1) to more urbanised downstream (Fig. 5c).

### Characterising the river resistome

We quantified the river water resistome by applying high-throughput qPCR with 283 ARG, eight transposase and four integron primers. For this paper, we define the sum of transposase genes plus integron genes as MGEs, although we recognise that this is only an estimate based on the limited number of genes we quantified. In total, 211 ARGs (~ 75% of those assayed) were detected in the river catchment with 70 ARGs (25% of assay) shared between all river water samples (n = 38 samples). All 12 MGEs were measured at least once in the sample with eight MGEs (75% of assay) shared across all samples (n = 38) (Additional file 1: Table S3). Detected ARGs encoded resistance to eight classes of antibiotics, with β-lactam resistance being the most common (45 detected/52 in the assay) (Additional file 1: Table S3).

Summarizing ARGs and MGEs, their detected numbers (number of ARGs or MGEs), river water concentrations (log_10_ ARG or MGE copies/mL) and cell concentrations (ARG or MGE copies/cell) all significantly increased from upstream (S1) to downstream (S8) (Welch’s t-test with *P* < 0.05 and large Cohen’s D effect size < − 0.8, Additional file 1: Table S4) with the Melana tributary frequently having the highest ARG and MGE concentrations (Additional file 1: Fig. S6, Additional file 1: Table S5). River water ARG concentrations increased more than two log_10_ steps along the catchment with ARG copy numbers per cell increasing from 0.1 copies/cell upstream to 2.2 copies/cell downstream (Additional file 1: Fig. S6, Additional file 1: Table S5).

The most abundant ARGs in the catchment encoded resistance against sulphonamides (*sul2*), aminoglycosides (*aadA1, aadA2*,), β-lactams (*blaOXA10*) and for non-specific efflux pumps (*qacEdelta1*, *qacH*) with their mean concentrations ranging between 1 × 10^7^ to 2 × 10^6^ gene copies/mL river water (Additional file 1: Table S6).

**Fig. 6:**
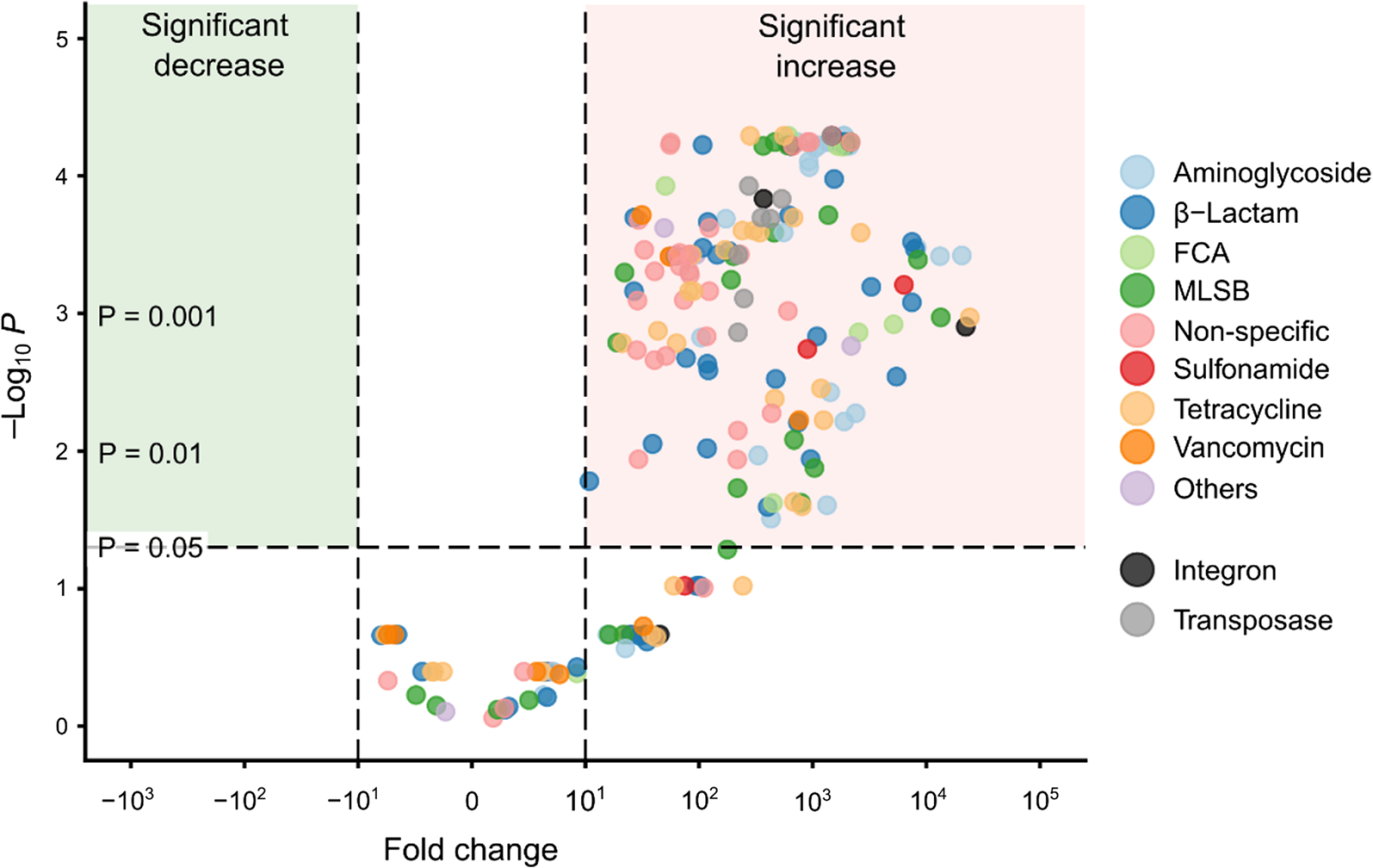
Volcano plot displaying ARG and MGE log_10_ fold river water concentration changes between upstream (S1) and downstream (S8). Statistical significance calculated with the Welch’s t-test, applying Benjamini–Hochberg P adjustment. Fold change calculated by subtracting mean ARG or MGE river water concentration for S1 (n = 5) from S8 (n = 5)

To assess the resistome changes along the river, we plotted ARG and MGE log_10_ fold river water concentration changes from up- to downstream (S1 to S8) against statistical significance in a volcano plot (Fig. 6). 146 ARG and MGE concentrations increased significantly at least tenfold between up- and downstream (Welch’s t-test, Benjamini–Hochberg adjusted *P* < 0.05). Four ARGs encoding for aminoglycoside, MLSB and tetracycline resistance and integron 3 increased more than four log_10_ steps from up- to downstream (Fig. 6).

### Network analysis of microbiomes and resistomes

**Fig. 7:**
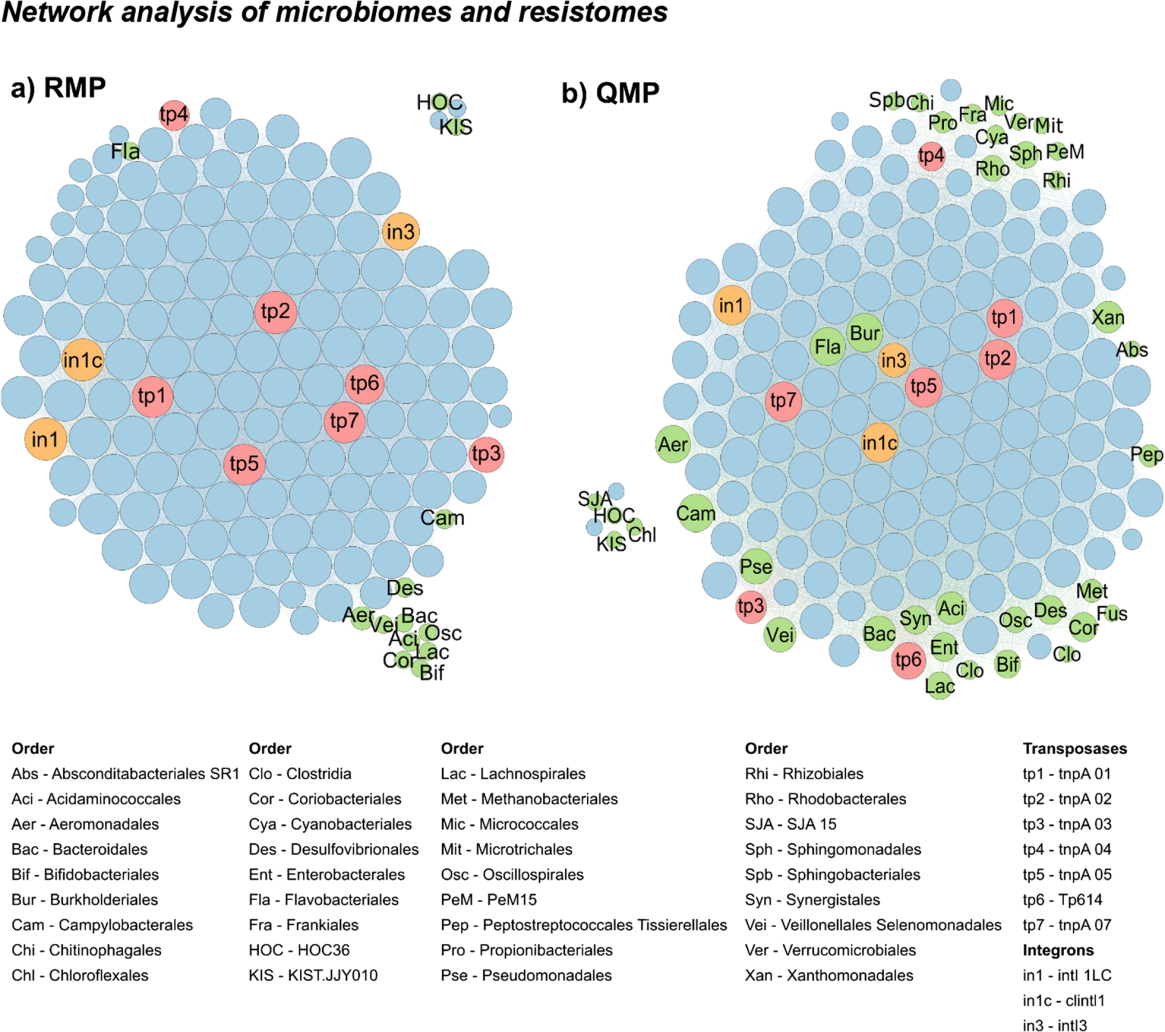
Network analysis based on relative (RMP; **a**) and quantitative (QMP; **b**) microbiome profiling, revealing co-occurrence patterns among ARGs (blue circles), MGEs (red and orange circles) and taxa at order level (green circles). A connection represents a strong (Spearman’s ρ > 0.8) and significant (*P* < 0.01, adjusted with Benjamini Hochberg) correlation. The size of each node is proportional to the number of connections (= degree). Only nodes with at least three other connections are shown. For more details, see Additional file 1: Table S7

Network analysis has been proposed to explore the associations between microbiomes and resistomes, but to date, such networks have been either based on relative values [10] or semi-quantitative data (relative NGS data for microbiomes and absolute HT-qPCR for resistomes [11], see Fig. 7a). Combining QMP (rather than RMP) with HT-qPCR data allows one to more fully compose the quantitative networks (Fig. 7b), overcoming negative correlation biases and spurious associations reported for relative abundance co-occurrence networks [16]. Based on the absolute taxa abundance data, the QMP network had a higher number of nodes and edges with a higher average node connectivity (= average degree) than the RMP network (Fig. 7, Additional file 1: Table S7). While for the QMP network, 36 taxa at order level had strong correlations (Spearman’s ρ > 0.8 and *P* < 0.01) with at least three other nodes, this was only the case for 13 taxa in the RMP network (Fig. 7, Additional file 1: Table S7).

For the QMP network, the most connected ARGs, transposases, and integrons were *blaOXA10* (152 degrees), *tnpA 02* (147 degrees) and clinical integron 1 (*clintl1*; 146 degrees), respectively (Fig. 7b). The most correlating taxa belonged to the order of *Burkholderiales* (141 degrees), *Flavobacteriales* (135 degrees) and *Campylobacterales* (134 degrees), indicating that these bacteria might be frequent hosts of ARGs, and/or that these bacteria came from a similar source to the ARGs and MGEs (Fig. 7b). While these correlations do not replace further monitoring, they help in hypothesis formulation, addressing better-grounded research questions [14].

## Discussion

Our understanding of complex environmental microbiomes has been hindered by overly relying on relative abundance data and inconsistent definitions of diversity in describing microbial changes. This hampers the ability of environmental researchers to reliably link microbiome and resistome changes in the investigation of AR fate and spread, and other practical questions [7], such as providing quantitative data for QMRAs—a crucial knowledge gap for assessing environmental AR exposure risk.

To date, few papers have reported absolute taxa abundances [20, 40, 75] and, to our knowledge, only one human study [17] used rarefaction to make sampling depths equal prior to multiplying the relative taxa abundances with cell concentrations. While this normalization step removes sequencing information for 'over-sequenced' samples (here upstream S1), it is necessary to allow a reliable comparison of microbial diversity, especially when cell counts vary widely across samples (here 100-fold). Only after sampling depth correction in QMP, did we find diversity to have increased significantly in the catchment from rural up- to urban downstream; this critical observation was not possible using the RMP approach.

Despite environmental QMP not addressing all known biases in microbiome research, it allows more accurate and easier absolute quantification of microbiota variation. In environmental studies, cell counts are routinely measured and QMP can be conducted at no extra cost, requiring little bioinformatic workflow adjustments. In this study, absolute taxa abundance data allowed to explore environmental microbiome and resistome interactions, overcoming biases related to relative taxa abundance data. Once bias is reduced, one then has more exact numerical data for QMRA calculations, which is essential for statistical and other analysis with parallel health and other end-point data within a QMRA.

Several methods are available to estimate cell counts, and one must consider the benefits and limitations of each option relative to absolute taxa abundance calculations. Here, we estimated cell counts by dividing 16S rRNA qPCR concentrations with the average 16S rRNA GCN per bacterium (4.1, [39]). This is a generalised approach because 16S rRNA GCN can vary greatly across cells. Measuring total cells using flow cytometry is another possible option [17, 20]. Flow cytometry protocols are available for almost all environmental compartments (e.g. wastewater [76], biofilms [77] or seawater [78]) to optimise cell detection.

Conversely, parallel qPCR quantification of the same products as NGS is an option that might reduce bias from non-genetic methods (e.g., flow cytometry of cells vs. qPCR of 16S rRNA). Recent advances now also allow the quantification of viable cells with digital PCR [79, 80]. When using qPCR, the same primer region should be targeted to estimate cell counts and assess the microbiome. Further qPCR bias could be reduced by diluting sample DNA instead of normalising to a DNA concentration. We recommend further research to compare cell concentration measurements for QMP.

Diversity has been defined in so many different ways that its ability to transfer accurate information on microbial community changes, e.g. due to human impact, is compromised [24]. Jost and Chao (2020) introduced the analogy that diversity indices (e.g. Shannon or Simpson index) are connected to diversity in the same manner as a sphere’s diameter is connected to its volume. While the diameter is an index of the sphere’s volume, it is not the volume itself. They state that using the diameter instead of volume in engineering calculations would result in chaos, but this is what biologists are currently doing with diversity indices [74]. Shannon and Simpson index are useful diversity indices with an important role in ecology, but their values provide information on uncertainty and probability, respectively, rather than measuring diversity [23]. The Hill number framework provides a better and more unified approach to calculate and compare microbial diversities across environmental compartments, especially where the parameter q can be used to modulate the sensitivity towards abundant versus rare ASVs.

Depending on the study purpose, scientists might choose to calculate Hill numbers for several q for an in-depth diversity analysis (as performed here) or for one q value only. To define a core microbiome or when rare ASVs are considered untrustworthy due to technical bias (e.g. PCR or sequencing errors), q = 2 could be chosen to put more weight on abundant ASVs and results could be interpreted as effective number of dominant ASVs in the system [25, 30]. In contrast, when the rarest ASVs are as important as the most abundant ASVs, for example for conservation purposes, q = 0 could be chosen [30]. The recently published R hilldiv package [33] enables DNA-based diversity calculations with Hill numbers.

In this study, we observed an increase in diversity and decrease in evenness along the river from a less polluted upstream to a more polluted downstream. Environmental AR increased along the river as indicated by the enrichment of ARGs and MGEs. Resistome concentrations in the heavily urbanized Melana tributary were often higher than in the river itself. The increase in diversity, together with the increasing levels of cell counts, ARGs and MGEs in this rural-to-urban catchment are likely caused by insufficiently treated sewage entering the river (as previously shown in our sub-study of the same catchment [36] and in a different study covering the same area [81]). The most abundant ASVs for this catchment were *Cloacibacterium*, *Acinetobacter*, C39 (genus level) and *Comamonadaceae* (family level), also common in wastewater-impacted water bodies in China and India [82–84]. Comparing co-occurrence networks of absolute taxa with absolute ARG and MGE data allowed proposing hypothesis of possible taxa harbouring AR to be further investigated in experimental studies.

## Conclusions

This study shows the straightforward and easy implementation of a quantitative microbial profiling approach and intuitive diversity characterisation with Hill numbers. We recommend our new combined approach to become the norm for future environmental microbiome (and resistome) research, especially to underpin improved QMRAs. Only when such methods are employed will environmental AR studies become more quantitative and truly comparable.

## Supplementary Information

**Additional file 1.** Supplementary information.

## Data Availability

Raw amplicon sequencing data that support the findings of this study have been deposited in European Nucleotide Archive with study accession number PRJEB42314. All other data can be accessed through the Center for Open Science, OSF (Ott, Amelie. 2021. 'Monitoring and Modelling of Antibiotic Resistance in Southeast Asian Rivers.' OSF. https://osf.io/gcpky/?view_only=90e614c2c6b64483aa503694af113789).
