# Supplementary material for: Improved quantitative microbiome profiling for environmental antibiotic resistance surveillance
Source: Environ Microbiome. 2021 Nov 18;16:21. doi: 10.1186/s40793-021-00391-0 (PMC8600772; doi:10.1186/s40793-021-00391-0)
Supplement: Supplementary file 1 — Additional file 1. Supplementary information. [file 40793_2021_391_MOESM1_ESM.docx]

**Supplementary Material**

**Improved quantitative microbiome profiling for environmental antibiotic resistance surveillance**

Amelie Ott^1^, Marcos Quintela-Baluja^1^, Andrew M. Zealand^1^, Greg O'Donnell^1^, Mohd Ridza Mohd Haniffah^2^, and David W. Graham^1*^

^1^ Newcastle University, School of Engineering, Newcastle upon Tyne, NE1 7RU, UK

^2^ Universiti Teknologi Malaysia, Jalan Iman, 81310 Skudai, Johor, Malaysia

^*^Corresponding author: Prof David W. Graham

School of Engineering

Cassie Building

Newcastle University

Newcastle upon Tyne

United Kingdom NE1 7RU

E-mail: [david.graham@newcastle.ac.uk](mailto:david.graham@newcastle.ac.uk)

Supplementary Figures


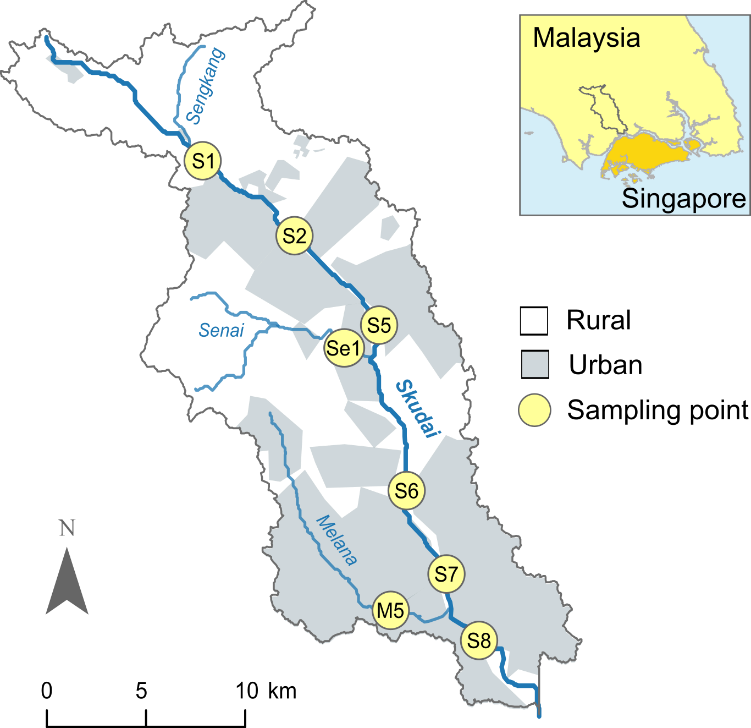


**Additional file 1: Figure S1.** Skudai catchment in Malaysia with sampling points.[1]


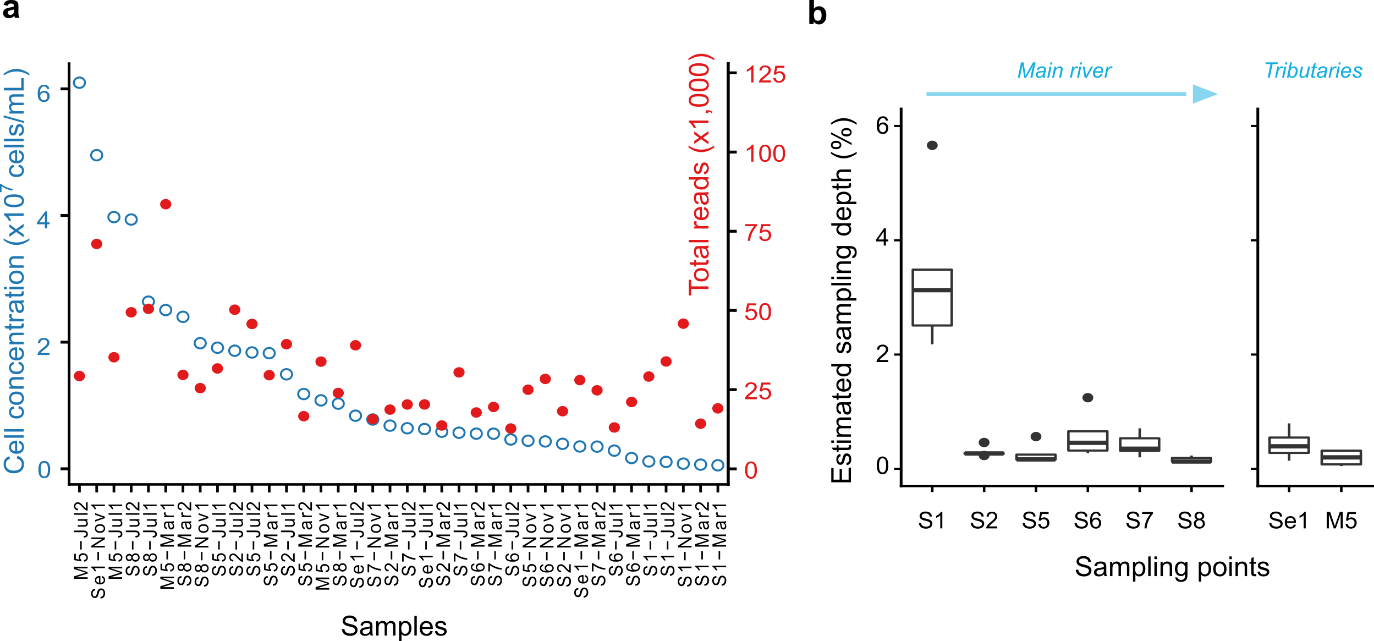


**Additional file 1: Figure S2.** Cell concentration and reads per sample (a) and sampling depth per sampling point (b). For (a), Samples ranked according to decreasing cell concentration. Cell concentration and reads per sample correlated moderately (n = 38, Spearman ρ = 0.52, P = 0.0007528). For sampling depth (b), reads per sample (=sequencing depth) was divided by cell concentration. Data represented is based on five biological replicates for the main river (S1, S2, S5, S6, S7, S8) and on four biological replicates for the tributaries (Se1, M5). Box-plot elements are defined as center line (median), box limits (upper and lower quartiles), whiskers (1.5x interquartile range) and points (outliers).


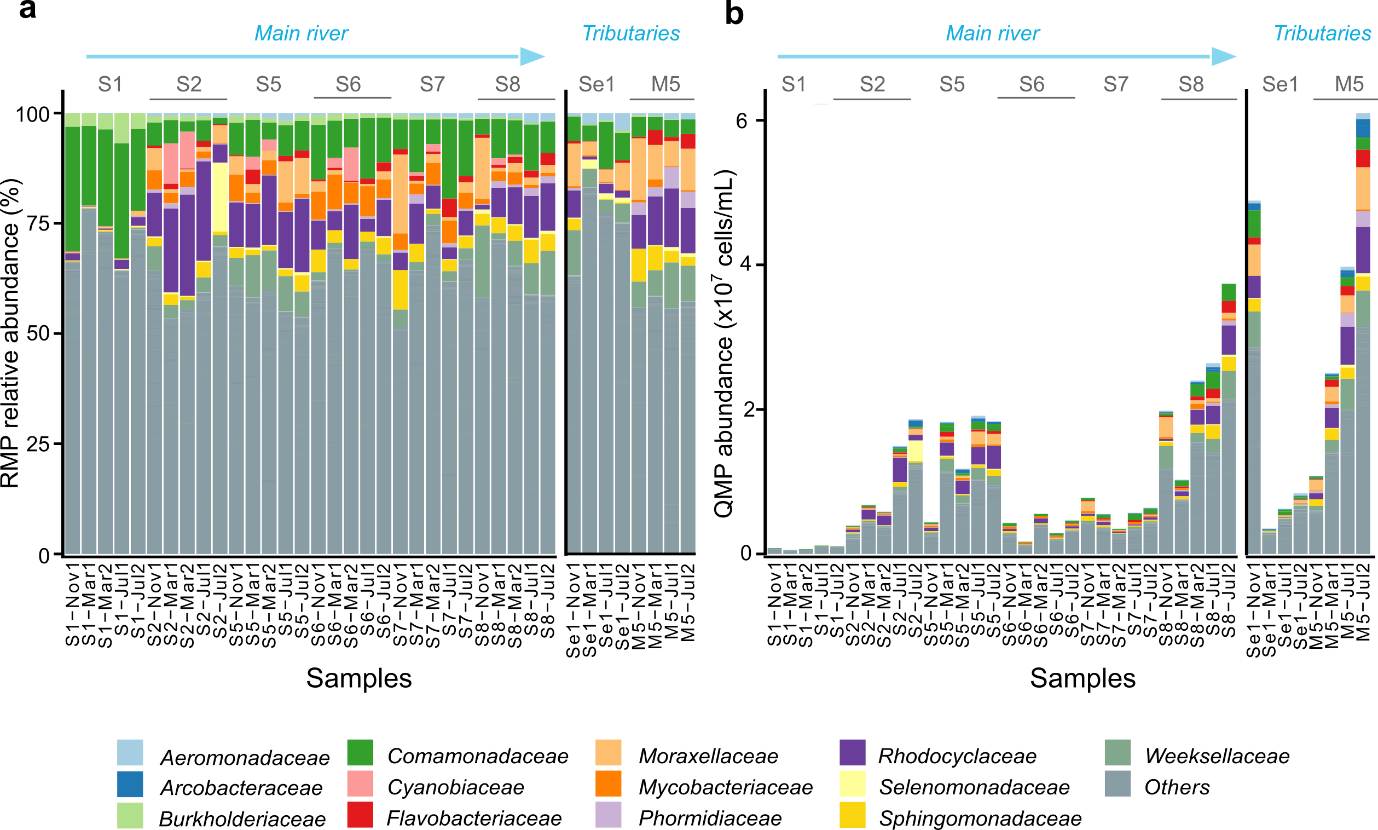


**Additional file 1: Figure S3.** Barplots showing the 20 most abundant ASVs grouped into families with remain pooled into 'Other' for the relative (RMP; a) and quantitative (QMP[2]; b) microbiome profiling approach. Data represented (n = 38) is based on five biological replicates for the main river (S1, S2, S5, S6, S7, S8) and on four biological replicates for the tributaries (Se1, M5).


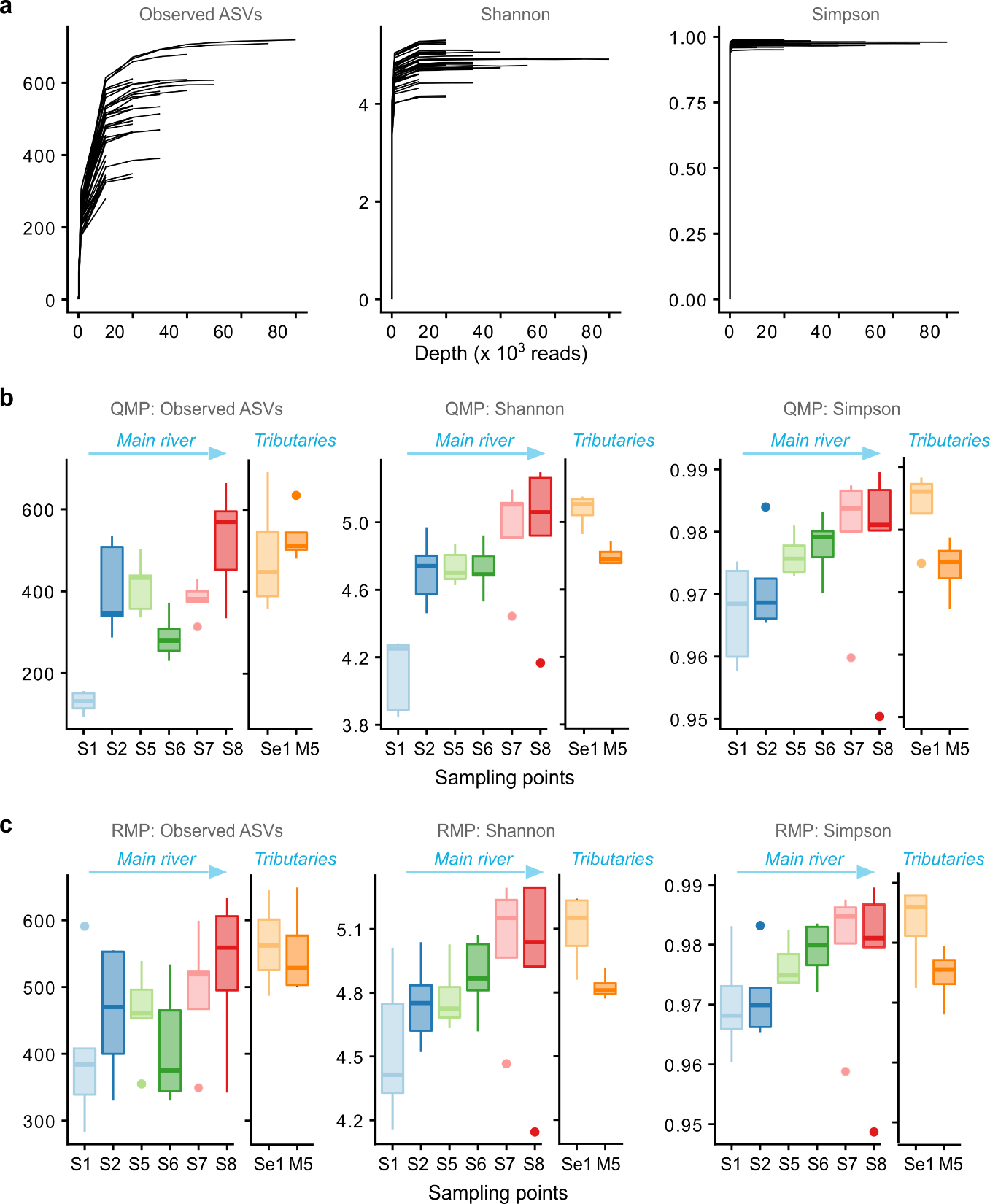


**Additional file 1: Figure S4.** Rarefaction curves (a) and alpha diversity indices (richness, Shannon index and Simpson index) for the QMP (b) and RMP (c) dataset. Data represented (n = 38) is based on five biological replicates for the main river (S1, S2, S5, S6, S7, S8) and on four biological replicates for the tributaries (Se1, M5). Box-plot elements are defined as center line (median), box limits (upper and lower quartiles), whiskers (1.5x interquartile range) and points (outliers).


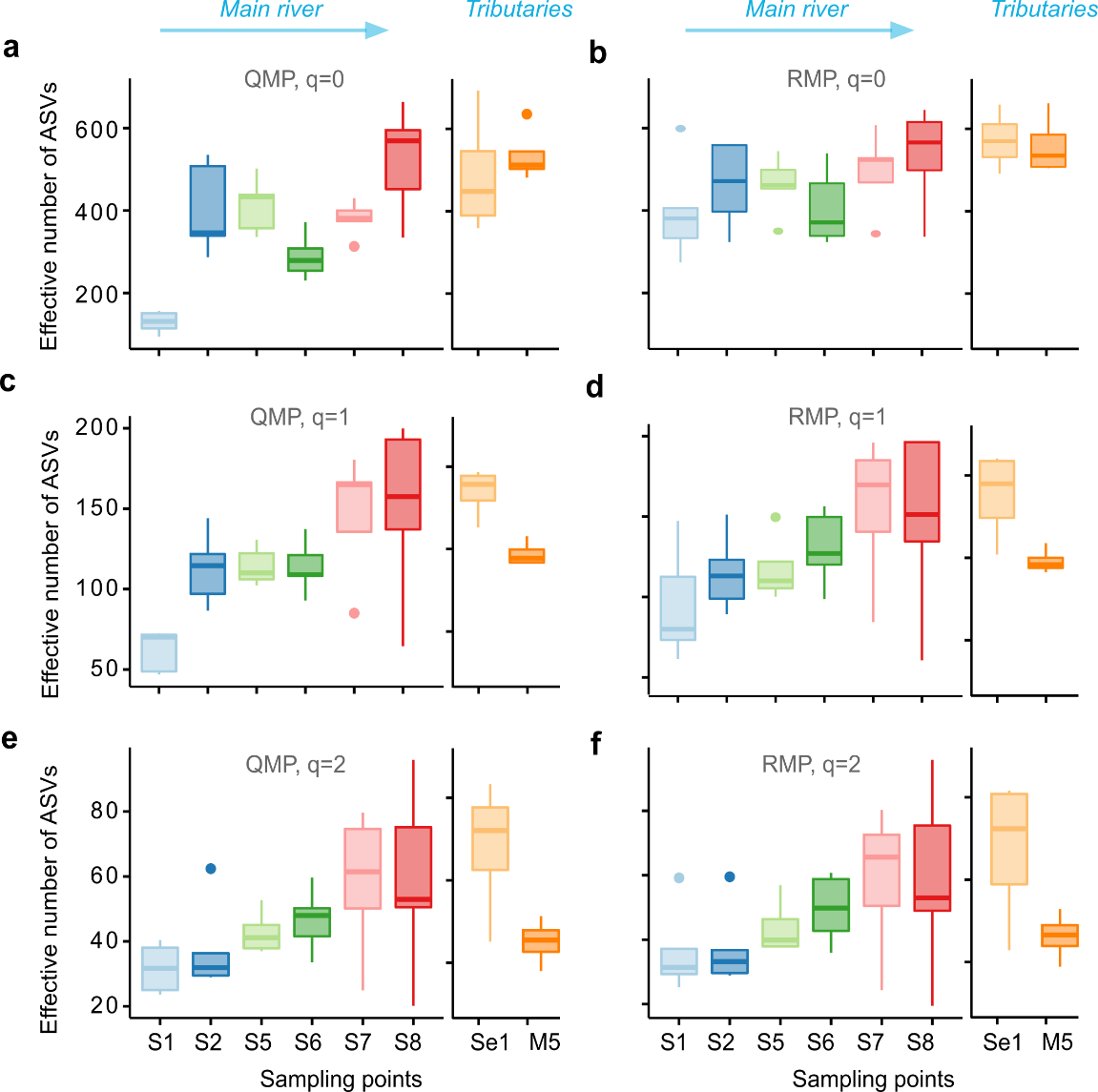


**Additional file 1: Figure S5.** Hill number α-diversities per sampling point for q = 0, q = 1 and q = 2 for the quantitative microbiome profiling (QMP) approach (a, c, e) and the relative microbiome profiling (RMP) approach (b, d, f) [3, 4]. Data represented (n = 38) is based on five biological replicates for the main river (S1, S2, S5, S6, S7, S8) and on four biological replicates for the tributaries (Se1, M5). Box-plot elements are defined as center line (median), box limits (upper and lower quartiles), whiskers (1.5x interquartile range) and points (outliers).


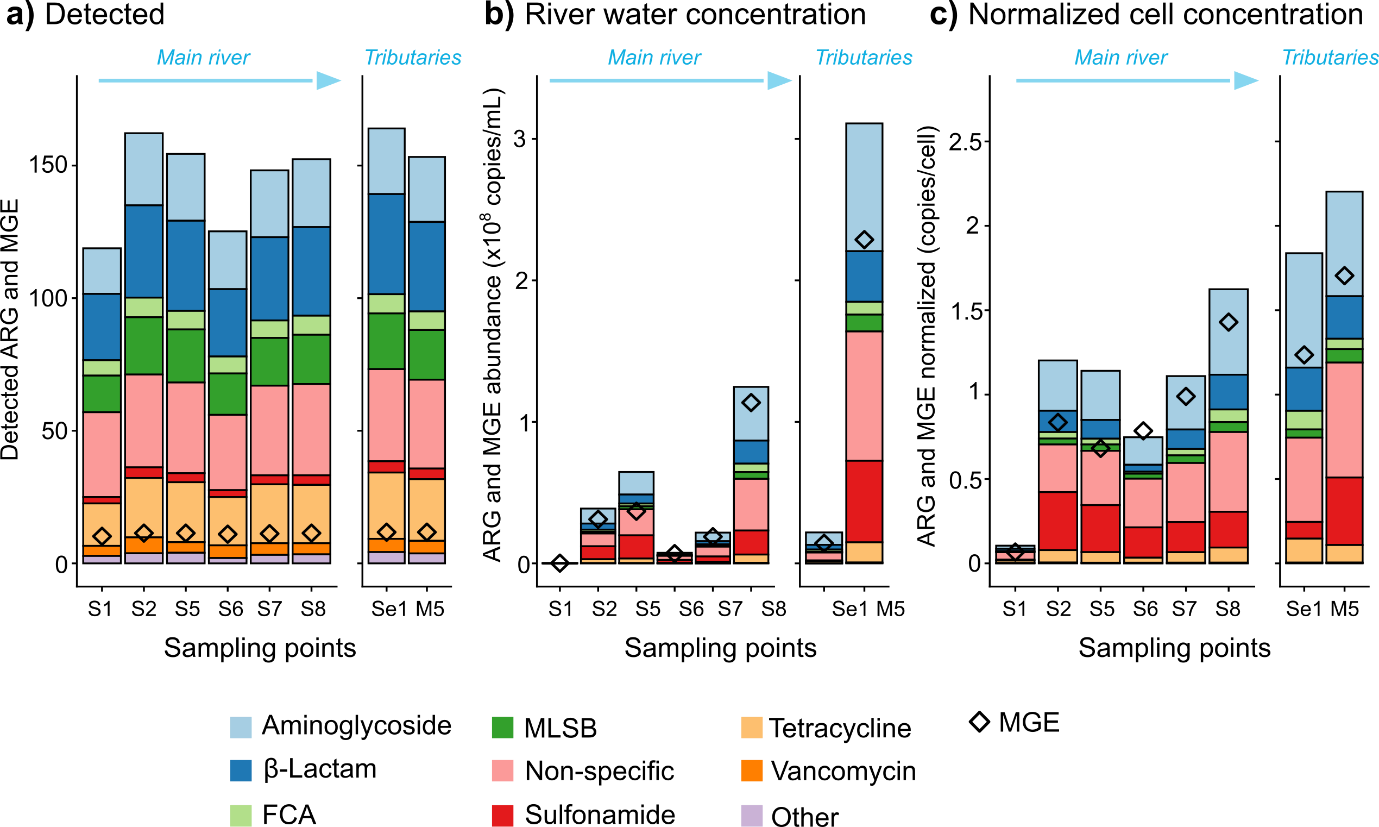


**Additional file 1: Figure S6.** ARG and MGE numbers (a), river water concentrations (b) and normalized cell concentration (c) detected with HT-qPCR per sampling point in the Skudai catchment. Mean data represented is based on five biological replicates for the main river (S1, S2, S5, S6, S7, S8) and on four biological replicates for the tributaries (Se1, M5).

Supplementary Tables

**Additional file 1: Table S1.** List of the 20 most abundant ASVs classified to genus level based on the QMP and RMP approach. For genus level, numbers in brackets were added to differentiate ASVs with the same genus. where grey colouring for the QMP or RMP listing highlights ASVs not present in the RMP or QMP listing, respectively. * indicates where ASV rank order is the same in the QMP and RMP listing. ND = not classified to genus level.

| Top 20 ASVs based QMP approach | | | | | |
| --- | --- | --- | --- | --- | --- |
| ASV | Phylum | Class | Order | Family | Genus |
| 1 | Bacteroidota | Bacteroidia | Flavobacteriales | *Weeksellaceae* | *Cloacibacterium (1)* |
| 2 | Proteobacteria | Gammaproteobacteria | Pseudomonadales | *Moraxellaceae* | *Acinetobacter (1)* |
| 3 | Proteobacteria | Gammaproteobacteria | Burkholderiales | *Rhodocyclaceae* | *C39 (1) ** |
| 4 | Proteobacteria | Gammaproteobacteria | Burkholderiales | *Comamonadaceae* | *ND (1)* |
| 5 | Proteobacteria | Alphaproteobacteria | Sphingomonadales | *Sphingomonadaceae* | *Novosphingobium (1)** |
| 6 | Proteobacteria | Gammaproteobacteria | Burkholderiales | *Rhodocyclaceae* | *C39 (2)** |
| 7 | Proteobacteria | Gammaproteobacteria | Burkholderiales | *Rhodocyclaceae* | *C39 (3)* |
| 8 | Bacteroidota | Bacteroidia | Flavobacteriales | *Flavobacteriaceae* | *Flavobacterium (1)* |
| 9 | Proteobacteria | Gammaproteobacteria | Burkholderiales | *Rhodocyclaceae* | *C39 (4)* |
| 10 | Cyanobacteria | Cyanobacteriia | Cyanobacteriales | *Phormidiaceae* | *Planktothrix_NIVA-CYA_15 (1)* |
| 11 | Actinobacteriota | Actinobacteria | Corynebacteriales | *Mycobacteriaceae* | *Mycobacterium (1)* |
| 12 | Proteobacteria | Gammaproteobacteria | Pseudomonadales | *Moraxellaceae* | *Acinetobacter (2)* |
| 13 | Proteobacteria | Gammaproteobacteria | Burkholderiales | *Comamonadaceae* | *ND (2)* |
| 14 | Proteobacteria | Gammaproteobacteria | Aeromonadales | *Aeromonadaceae* | *Tolumonas (1)* |
| 15 | Firmicutes | Negativicutes | Veillonellales-Selenomonadales | *Selenomonadaceae* | *ND (3)* |
| 16 | Campilobacterota | Campylobacteria | Campylobacterales | *Arcobacteraceae* | *Arcobacter (1)* |
| 17 | Campilobacterota | Campylobacteria | Campylobacterales | *Arcobacteraceae* | *Pseudarcobacter (1)* |
| 18 | Proteobacteria | Alphaproteobacteria | Sphingomonadales | *Sphingomonadaceae* | *Novosphingobium (1)* |
| 19 | Proteobacteria | Gammaproteobacteria | Burkholderiales | *Comamonadaceae* | *Aquabacterium (1)* |
| 20 | Bacteroidota | Bacteroidia | Flavobacteriales | *Flavobacteriaceae* | *Flavobacterium (1)* |
| Top 20 ASVs based RMP approach | | | | | |
| ASV | Phylum | Class | Order | Family | Genus |
| 1 | Proteobacteria | Gammaproteobacteria | Burkholderiales | *Comamonadaceae* | *ND (1)* |
| 2 | Bacteroidota | Bacteroidia | Flavobacteriales | *Weeksellaceae* | *Cloacibacterium (1)* |
| 3 | Proteobacteria | Gammaproteobacteria | Burkholderiales | *Rhodocyclaceae* | *C39 (1)** |
| 4 | Proteobacteria | Gammaproteobacteria | Pseudomonadales | *Moraxellaceae* | *Acinetobacter (1)* |
| 5 | Proteobacteria | Alphaproteobacteria | Sphingomonadales | *Sphingomonadaceae* | *Novosphingobium (1)** |
| 6 | Proteobacteria | Gammaproteobacteria | Burkholderiales | *Rhodocyclaceae* | *C39 (2)** |
| 7 | Actinobacteriota | Actinobacteria | Corynebacteriales | *Mycobacteriaceae* | *Mycobacterium (1)* |
| 8 | Proteobacteria | Gammaproteobacteria | Burkholderiales | *Comamonadaceae* | *ND (4)* |
| 9 | Proteobacteria | Gammaproteobacteria | Burkholderiales | *Comamonadaceae* | *ND (5)* |
| 10 | Proteobacteria | Gammaproteobacteria | Burkholderiales | *Burkholderiaceae* | *Polynucleobacter (1)* |
| 11 | Proteobacteria | Gammaproteobacteria | Pseudomonadales | *Moraxellaceae* | *Acinetobacter (2)* |
| 12 | Proteobacteria | Gammaproteobacteria | Burkholderiales | *Comamonadaceae* | *ND (2)* |
| 13 | Proteobacteria | Gammaproteobacteria | Burkholderiales | *Rhodocyclaceae* | *C39 (3)* |
| 14 | Bacteroidota | Bacteroidia | Flavobacteriales | *Flavobacteriaceae* | *Flavobacterium (1)* |
| 15 | Cyanobacteria | Cyanobacteriia | Synechococcales | *Cyanobiaceae* | *Cyanobium_PCC-6307 (1)* |
| 16 | Proteobacteria | Gammaproteobacteria | Burkholderiales | *Rhodocyclaceae* | *C39 (4)* |
| 17 | Proteobacteria | Gammaproteobacteria | Aeromonadales | *Aeromonadaceae* | *Tolumonas (1)* |
| 18 | Cyanobacteria | Cyanobacteriia | Cyanobacteriales | *Phormidiaceae* | *Planktothrix_NIVA-CYA_15 (1)* |
| 19 | Proteobacteria | Gammaproteobacteria | Burkholderiales | *Comamonadaceae* | *ND (6)* |
| 20 | Firmicutes | Negativicutes | Veillonellales-Selenomonadales | *Selenomonadaceae* | *ND (3)* |

**Additional file 1: Table S2.** Results from the Welch’s t-test and Cohen’s D effect size calculations comparing Hill number diversities upstream (S1) and downstream (S8). Comparisons based on five biological replicates for each site. Statistical significance for P < 0.05 and large effect size for D < -0.8.

|  | Welch’s t-test | | | Cohen’s D effect size | | |
| --- | --- | --- | --- | --- | --- | --- |
|  | P value | Degrees of freedom | t value | Value | 95% confidence interval range | |
| QMP q=0 | 0.0021 | 4.31 | -6.62 | -4.19 | -6.80 | -1.58 |
| QMP q=1 | 0.0203 | 4.45 | -3.53 | -2.23 | -4.09 | -0.38 |
| QMP q=2 | 0.0993 | 4.56 | -2.06 | -1.31 | -2.91 | 0.30 |
| RMP q=0 | 0.1245 | 8.00 | -1.72 | -1.09 | -2.65 | 0.48 |
| RMP q=1 | 0.1175 | 6.71 | -1.80 | -1.14 | -2.71 | 0.44 |
| RMP q=2 | 0.1747 | 5.65 | -1.55 | -0.98 | -2.53 | 0.56 |

**Additional file 1: Table S3.** ARG and MGE numbers included in the HT-qPCR assay, detected in each river water sample and maximum amount detected.

|  | Gene number included in HT-qPCR assay | Detected in each river water sample (n=38) | | Maximum amount detected in catchment | |
| --- | --- | --- | --- | --- | --- |
|  |  | Number | % of assay | Number | % of assay |
| ARGs | 283 | 70 | 25 | 211 | 75 |
| - Aminoglycoside | 36 | 13 | 36 | 31 | 86 |
| - β-Lactams | 52 | 14 | 27 | 45 | 87 |
| - FCA | 9 | 4 | 44 | 9 | 100 |
| - MLSB | 46 | 9 | 20 | 29 | 63 |
| - Non-specific | 51 | 14 | 27 | 39 | 76 |
| - Others | 11 | 1 | 9 | 8 | 73 |
| - Sulfonamide | 7 | 1 | 14 | 6 | 86 |
| - Tetracycline | 39 | 12 | 31 | 31 | 79 |
| - Vancomycin | 32 | 2 | 6 | 13 | 41 |
| MGEs | 12 | 9 | 75 | 12 | 100 |
| - Integrons | 4 | 2 | 50 | 4 | 100 |
| - Transposase | 8 | 7 | 88 | 8 | 100 |
| ALL | 578 | 79 | 14 | 223 | 39 |

**Additional file 1: Table S4.** Results from the Welch’s t-test and Cohen’s D effect size calculations comparing log_10_ ARG and MGE river water concentrations (gene copies/mL), ARG and MGE detected numbers, and ARG and MGE normalized cell concentrations for up-(S1) and downstream (S8). Comparisons based on five biological replicates for each site. Statistical significance for P < 0.05 and large effect size for D < -0.8.

|  | Welch’s t-test | | | Cohens D effect size | | |
| --- | --- | --- | --- | --- | --- | --- |
|  | P value | Degrees of freedom | t value | Exact value | 95% confidence interval range | |
| ARG log_10_ river water concentration (copies/mL) | <0.0001 | 6.91 | -11.75 | -7.43 | -11.53 | -3.33 |
| MGE log_10_ river water concentration (copies/mL) | <0.0001 | 7.19 | -12.07 | -7.63 | -11.83 | -3.44 |
| Detected ARGs (number) | 0.0015 | 7.06 | -5.01 | -3.17 | -5.36 | -0.98 |
| Detected MGEs (number) | 0.0057 | 7.69 | -3.79 | -2.40 | -4.31 | -0.49 |
| ARG normalized cell concentration (copies/cell) | 0.0035 | 4.10 | -6.06 | -3.83 | -6.29 | -1.38 |
| MGE normalized cell concentration (copies/cell) | 0.0071 | 4.03 | -5.06 | -3.20 | -5.40 | -1.00 |

**Additional file 1: Table S5.** ARG and MGE detected numbers, river water concentrations and normalized cell concentrations per sampling point in the Skudai catchment. Mean and standard deviations based on five biological replicates for the main river (S1, S2, S5, S6, S7, S8) and on four biological replicates for the tributaries (Se1, M5).

|  | Detected (number) | | River water concentration (copies/mL) | | Normalized cell concentration (copies/cell) | |
| --- | --- | --- | --- | --- | --- | --- |
|  | ARGs | MGEs | ARGs | MGEs | ARGs | MGEs |
| S1 | 118.8 ± 12.4 | 10.2 ± 0.4 | (1.8 ± 1.5) x 10^5^ | (1.2 ± 1) x 10^5^ | 0.1 ± 0.06 | 0.07 ± 0.04 |
| S2 | 162.2 ± 13.5 | 11.4 ± 0.5 | (3.4 ± 3.1) x 10^7^ | (2.6 ± 3.3) x 10^7^ | 1.2 ± 0.42 | 0.84 ± 0.61 |
| S5 | 154.4 ± 6.2 | 11.4 ± 0.9 | (5.4 ± 2.6) x 10^7^ | (3.1 ± 1.5) x 10^7^ | 1.14 ± 0.14 | 0.68 ± 0.14 |
| S6 | 125.2 ± 13.1 | 11.0 ± 0.0 | (8.6 ± 4.7) x 10^6^ | (8 ± 3.4) x 10^6^ | 0.75 ± 0.14 | 0.79 ± 0.19 |
| S7 | 148.2 ± 8.6 | 11.2 ± 0.4 | (2.2 ± 0.6) x 10^7^ | (2.1 ± 0.9) x 10^7^ | 1.11 ± 0.19 | 0.99 ± 0.28 |
| S8 | 152.4 ± 8.4 | 11.4 ± 0.5 | (1.1 ± 0.8) x 10^8^ | (1 ± 0.8) x 10^8^ | 1.62 ± 0.56 | 1.43 ± 0.6 |
| Se1 | 164.0 ± 3.7 | 11.8 ± 0.5 | (5.7 ± 7) x 10^7^ | (3.9 ± 5.1) x 10^7^ | 1.84 ± 0.57 | 1.24 ± 0.27 |
| M5 | 153.3 ± 9.3 | 11.8 ± 0.5 | (2.4 ± 2.5) x 10^8^ | (1.8 ± 1.9) x 10^8^ | 2.2 ± 1.22 | 1.7 ± 0.89 |

**Additional file 1: Table S6.** Ten most abundant ARGs in the Skudai catchment based on the mean river water concentration (n = 38).

| Gene name | Classification | Mechanism | Mean ARG copies/mL | Standard deviation ARG copies/mL |
| --- | --- | --- | --- | --- |
| sul2 | Sulfonamide | Cellular protection | 1.1 x 10^7^ | 1.8 x 10^7^ |
| qacEdelta1_02 | Non-specific | Efflux pump | 7.2 x 10^6^ | 1.3 x 10^7^ |
| qacEdelta1_01 | Non-specific | Efflux pump | 7 x 10^6^ | 1.3 x 10^7^ |
| aadA2_03 | Aminoglycoside | Antibiotic deactivate | 4.2 x 10^6^ | 8.1 x 10^6^ |
| aadA1 | Aminoglycoside | Antibiotic deactivate | 3.2 x 10^6^ | 6 x 10^6^ |
| qacH_02 | Non-specific | Efflux pump | 2.3 x 10^6^ | 3.6 x 10^6^ |
| aadA2_01 | Aminoglycoside | Antibiotic deactivate | 2.1 x 10^6^ | 4.5 x 10^6^ |
| aadA2_02 | Aminoglycoside | Antibiotic deactivate | 2. x 10^6^ | 4.4 x 10^6^ |
| blaOXA10_01 | β-Lactam | Antibiotic deactivate | 2. x 10^6^ | 3.6 x 10^6^ |
| blaOXA10_02 | β-Lactam | Antibiotic deactivate | 1.7 x 10^6^ | 2.9 x 10^6^ |

**Additional file 1: Table S7.** Properties for the ARG, MGE and taxa (order level) networks based on the relative microbiome profiling (RMP) approach (see Figure 1‑7a) and quantitative microbiome profiling (QMP) approach (see Figure 1‑7b). Only nodes with at least three other connections are shown.

|  | RMP | QMP |
| --- | --- | --- |
| Nodes | 153 | 176 |
| - ARGs | 130 | 130 |
| - Transposase | 7 | 7 |
| - Integrons | 3 | 3 |
| - Taxa | 13 | 36 |
| Edges | 7690 | 9455 |
| Network diameter (maximum distance between edges) | 5 | 3 |
| Average path length | 1.4 | 1.37 |
| Average degree (node connectivity) | 100.523 | 107.443 |
| Graph density | 0.661 | 0.614 |
| Modularity index | 0.072 | 0.094 |

**Additional file 1: Table S8.** HT-qPCR primers.[5, 6] FCA = (flor)/(chlor)/(am)phenicol. MLSB = macrolides, lincosamides and streptogramin B.

| Assay ID | Forward Primer | Reverse Primer | Classification | Mechanism |
| --- | --- | --- | --- | --- |
| *16S rRNA* | GGGTTGCGCTCGTTGC | ATGGYTGTCGTCAGCTCGTG |  |  |
| *catA1* | GGGTGAGTTTCACCAGTTTTGATT | CACCTTGTCGCCTTGCGTATA | FCA | deactivate |
| *catB3* | GCACTCGATGCCTTCCAAAA | AGAGCCGATCCAAACGTCAT |  | deactivate |
| *catB8* | CACTCGACGCCTTCCAAAG | CCGAGCCTATCCAGACATCATT |  | deactivate |
| *cfr* | GCAAAATTCAGAGCAAGTTACGAA | AAAATGACTCCCAACCTGCTTTAT |  | deactivate |
| *cmlA1-01* | TAGGAAGCATCGGAACGTTGAT | CAGACCGAGCACGACTGTTG |  | efflux |
| *cmlA1-02* | AGGAAGCATCGGAACGTTGA | ACAGACCGAGCACGACTGTTG |  | efflux |
| *cmx(A)* | GCGATCGCCATCCTCTGT | TCGACACGGAGCCTTGGT |  | efflux |
| *floR* | ATTGTCTTCACGGTGTCCGTTA | CCGCGATGTCGTCGAACT |  | efflux |
| *qnrA* | AGGATTTCTCACGCCAGGATT | CCGCTTTCAATGAAACTGCAA |  | unknown |
| *aac* | CCCTGCGTTGTGGCTATGT | TTGGCCACGCCAATCC | Amino- glycoside | deactivate |
| *aac(6')I1* | GACCGGATTAAGGCCGATG | CTTGCCTTGATATTCAGTTTTTATAACCA |  | deactivate |
| *aac(6')-Ib(aka aacA4)-02* | CGTCGCCGAGCAACTTG | CGGTACCTTGCCTCTCAAACC |  | deactivate |
| *aac(6')-Ib(aka aacA4)-01* | GTTTGAGAGGCAAGGTACCGTAA | GAATGCCTGGCGTGTTTGA |  | deactivate |
| *aac(6')-Ib(aka aacA4)-03* | AGAAGCACGCCCGACACTT | GCTCTCCATTCAGCATTGCA |  | deactivate |
| *aac(6')-II* | CGACCCGACTCCGAACAA | GCACGAATCCTGCCTTCTCA |  | deactivate |
| *aac(6')-Iy* | GCTTTGCGGATGCCTCAAT | GGAGAACAAAAATACCTTCAAGGAAA |  | deactivate |
| *aacA/aphD* | AGAGCCTTGGGAAGATGAAGTTT | TTGATCCATACCATAGACTATCTCATCA |  | deactivate |
| *aacC* | CGTCACTTATTCGATGCCCTTAC | GTCGGGCGCGGCATA |  | deactivate |
| *aacC1* | GGTCGTGAGTTCGGAGACGTA | GCAAGTTCCCGAGGTAATCG |  | deactivate |
| *aacC2* | ACGGCATTCTCGATTGCTTT | CCGAGCTTCACGTAAGCATTT |  | deactivate |
| *aacC4* | CGGCGTGGGACACGAT | AGGGAACCTTTGCCATCAACT |  | deactivate |
| *aadA-01* | GTTGTGCACGACGACATCATT | GGCTCGAAGATACCTGCAAGAA |  | deactivate |
| *aadA-02* | CGAGATTCTCCGCGCTGTA | GCTGCCATTCTCCAAATTGC |  | deactivate |
| *aadA1* | AGCTAAGCGCGAACTGCAAT | TGGCTCGAAGATACCTGCAA |  | deactivate |
| *aadA-1-01* | AAAAGCCCGAAGAGGAACTTG | CATCTTTCACAAAGATGTTGCTGTCT |  | deactivate |
| *aadA-1-02* | CGGAATTGAAAAAACTGATCGAA | ATACCGGCTGTCCGTCATTT |  | deactivate |
| *aadA2-01* | ACGGCTCCGCAGTGGAT | GGCCACAGTAACCAACAAATCA |  | deactivate |
| *aadA2-02* | CTTGTCGTGCATGACGACATC | TCGAAGATACCCGCAAGAATG |  | deactivate |
| *aadA2-03* | CAATGACATTCTTGCGGGTATC | GACCTACCAAGGCAACGCTATG |  | deactivate |
| *aadA5-01* | ATCACGATCTTGCGATTTTGCT | CTGCGGATGGGCCTAGAAG |  | deactivate |
| *aadA5-02* | GTTCTTGCTCTTGCTCGCATT | GATGCTCGGCAGGCAAAC |  | deactivate |
| *aadA9-01* | CGCGGCAAGCCTATCTTG | CAAATCAGCGACCGCAGACT |  | deactivate |
| *aadA9-02* | GGATGCACGCTTGGATGAA | CCTCTAGCGGCCGGAGTATT |  | deactivate |
| *aadD* | CCGACAACATTTCTACCATCCTT | ACCGAAGCGCTCGTCGTATA |  | deactivate |
| *aadE* | TACCTTATTGCCCTTGGAAGAGTTA | GGAACTATGTCCCTTTTAATTCTACAATCT |  | deactivate |
| *aph* | TTTCAGCAAGTGGATCATGTTAAAAT | CCAAGCTGTTTCCACTGTTTTTC |  | deactivate |
| *aph(2')-Id-02* | TAAGGATATACCGACAGTTTTGGAAA | TTTAATCCCTCTTCATACCAATCCATA |  | deactivate |
| *aph(2')-Id-01* | TGAGCAGTATCATAAGTTGAGTGAAAAG | GACAGAACAATCAATCTCTATGGAATG |  | deactivate |
| *aph6ia* | CCCATCCCATGTGTAAGGAAA | GCCACCGCTTCTGCTGTAC |  | deactivate |
| *aphA1(aka kanR)* | TGAACAAGTCTGGAAAGAAATGCA | CCTATTAATTTCCCCTCGTCAAAAA |  | deactivate |
| *spcN-01* | AAAAGTTCGATGAAACACGCCTAT | TCCAGTGGTAGTCCCCGAATC |  | deactivate |
| *spcN-02* | CAGAATCTTCCTGAAAAGTTTGATGAA | CGCAGACACGCCGAATC |  | deactivate |
| *str* | AATGAGTTTTGGAGTGTCTCAACGTA | AATCAAAACCCCTATTAAAGCCAAT |  | deactivate |
| *strA* | CCGGTGGCATTTGAGAAAAA | GTGGCTCAACCTGCGAAAAG |  | deactivate |
| *strB* | GCTCGGTCGTGAGAACAATCT | CAATTTCGGTCGCCTGGTAGT |  | deactivate |
| *ampC/blaDHA* | TGGCCGCAGCAGAAAGA | CCGTTTTATGCACCCAGGAA | β-Lactam | deactivate |
| *ampC-01* | TGGCGTATCGGGTCAATGT | CTCCACGGGCCAGTTGAG |  | deactivate |
| *ampC-02* | GCAGCACGCCCCGTAA | TGTACCCATGATGCGCGTACT |  | deactivate |
| *ampC-04* | TCCGGTGACGCGACAGA | CAGCACGCCGGTGAAAGT |  | deactivate |
| *ampC-05* | CTGTTCGAGCTGGGTTCTATAAGTAAA | CAGTATCTGGTCACCGGATCGT |  | deactivate |
| *ampC-06* | CCGCTCAAGCTGGACCATAC | CCATATCCTGCACGTTGGTTT |  | deactivate |
| *ampC-07* | CCGCCCAGAGCAAGGACTA | GCTCGACTTCACGCCGTAAG |  | deactivate |
| *ampC-09* | CAGCCGCTGATGAAAAAATATG | CAGCGAGCCCACTTCGA |  | deactivate |
| *bla1* | GCAAGTTGAAGCGAAAGAAAAGA | TACCAGTATCAATCGCATATACACCTAA |  | deactivate |
| *bla-ACC-1* | CACACAGCTGATGGCTTATCTAAAA | AATAAACGCGATGGGTTCCA |  | deactivate |
| *blaCMY* | CCGCGGCGAAATTAAGC | GCCACTGTTTGCCTGTCAGTT |  | deactivate |
| *blaCMY2-01* | AAAGCCTCATGGGTGCATAAA | ATAGCTTTTGTTTGCCAGCATCA |  | deactivate |
| *blaCMY2-02* | GCGAGCAGCCTGAAGCA | CGGATGGGCTTGTCCTCTT |  | deactivate |
| *blaCTX-M-04* | CTTGGCGTTGCGCTGAT | CGTTCATCGGCACGGTAGA |  | deactivate |
| *blaCTX-M-01* | GGAGGCGTGACGGCTTTT | TTCAGTGCGATCCAGACGAA |  | deactivate |
| *blaCTX-M-02* | GCCGCGGTGCTGAAGA | ATCGGATTATAGTTAACCAGGTCAGATTT |  | deactivate |
| *blaCTX-M-03* | CGATACCACCACGCCGTTA | GCATTGCCCAACGTCAGATT |  | deactivate |
| *blaCTX-M-05* | GCGATAACGTGGCGATGAAT | GTCGAGACGGAACGTTTCGT |  | deactivate |
| *blaCTX-M-06* | CACAGTTGGTGACGTGGCTTAA | CTCCGCTGCCGGTTTTATC |  | deactivate |
| *blaGES* | GCAATGTGCTCAACGTTCAAG | GTGCCTGAGTCAATTCTTTCAAAG |  | deactivate |
| *bla-L1* | CACCGGGTTACCAGCTGAAG | GCGAAGCTGCGCTTGTAGTC |  | deactivate |
| *blaMOX/blaCMY* | CTATGTCAATGTGCCGAAGCA | GGCTTGTCCTCTTTCGAATAGC |  | deactivate |
| *blaIMP-02* | AAGGCAGCATTTCCTCTCATTTT | GGATAGATCGAGAATTAAGCCACTCT |  | deactivate |
| *blaIMP-01* | AACACGGTTTGGTGGTTCTTGTA | GCGCTCCACAAACCAATTG |  | deactivate |
| *blaOCH* | GGCGACTTGCGCCGTAT | TTTTCTGCTCGGCCATGAG |  | deactivate |
| *blaOKP* | GCCGCCATCACCATGAG | GGTGACGTTGTCACCGATCTG |  | deactivate |
| *blaOXA1/blaOXA30* | CGGATGGTTTGAAGGGTTTATTAT | TCTTGGCTTTTATGCTTGATGTTAA |  | deactivate |
| *blaOXA10-01* | CGCAATTATCGGCCTAGAAACT | TTGGCTTTCCGTCCCATTT |  | deactivate |
| *blaOXA10-02* | CGCAATTATCGGCCTAGAAACT | TTGGCTTTCCGTCCCATTT |  | deactivate |
| *blaOXY* | CGTTCAGGCGGCAGGTT | GCCGCGATATAAGATTTGAGAATT |  | deactivate |
| *blaPAO* | CGCCGTACAACCGGTGAT | GAAGTAATGCGGTTCTCCTTTCA |  | deactivate |
| *blaPER* | TGCTGGTTGCTGTTTTTGTGA | CCTGCGCAATGATAGCTTCAT |  | deactivate |
| *blaPSE* | TTGTGACCTATTCCCCTGTAATAGAA | TGCGAAGCACGCATCATC |  | deactivate |
| *blaROB* | GCAAAGGCATGACGATTGC | CGCGCTGTTGTCGCTAAA |  | deactivate |
| *blaSFO* | CCGCCGCCATCCAGTA | GGGCCGCCAAGATGCT |  | deactivate |
| *blaSHV-01* | TCCCATGATGAGCACCTTTAAA | TTCGTCACCGGCATCCA |  | deactivate |
| *blaSHV-02* | CTTTCCCATGATGAGCACCTTT | TCCTGCTGGCGATAGTGGAT |  | deactivate |
| *blaTEM* | AGCATCTTACGGATGGCATGA | TCCTCCGATCGTTGTCAGAAGT |  | deactivate |
| *blaTLA* | ACACTTTGCCATTGCTGTTTATGT | TGCAAATTTCGGCAATAATCTTT |  | deactivate |
| *blaVEB* | CCCGATGCAAAGCGTTATG | GAAAGATTCCCTTTATCTATCTCAGACAA |  | deactivate |
| *blaVIM* | GCACTTCTCGCGGAGATTG | CGACGGTGATGCGTACGTT |  | deactivate |
| *blaZ* | GGAGATAAAGTAACAAATCCAGTTAGATATGA | TGCTTAATTTTCCATTTGCGATAAG |  | deactivate |
| *cepA* | AGTTGCGCAGAACAGTCCTCTT | TCGTATCTTGCCCGTCGATAAT |  | deactivate |
| *cfiA* | GCAGCGTTGCTGGACACA | GTTCGGGATAAACGTGGTGACT |  | deactivate |
| *cfxA* | TCATTCCTCGTTCAAGTTTTCAGA | TGCAGCACCAAGAGGAGATGT |  | deactivate |
| *cphA-01* | GCGAGCTGCACAAGCTGAT | CGGCCCAGTCGCTCTTC |  | deactivate |
| *cphA-02* | GTGCTGATGGCGAGTTTCTG | GGTGTGGTAGTTGGTGTTGATCAC |  | deactivate |
| *fox5* | GGTTTGCCGCTGCAGTTC | GCGGCCAGGTGACCAA |  | deactivate |
| *mecA* | GGTTACGGACAAGGTGAAATACTGAT | TGTCTTTTAATAAGTGAGGTGCGTTAATA |  | protection |
| *NDM1* | ATTAGCCGCTGCATTGAT | CATGTCGAGATAGGAAGTG |  | deactivate |
| *pbp* | CCGGTGCCATTGGTTTAGA | AAAATAGCCGCCCCAAGATT |  | protection |
| *pbp2x* | TTTCATAAGTATCTGGACATGGAAGAA | CCAAAGGAAACTTGCTTGAGATTAG |  | protection |
| *Pbp5* | GGCGAACTTCTAATTAATCCTATCCA | CGCCGATGACATTCTTCTTATCTT |  | protection |
| *penA* | AGACGGTAACGTATAACTTTTTGAAAGA | GCGTGTAGCCGGCAATG |  | protection |
| *intI-1(clinic) = clint1* | CGAACGAGTGGCGGAGGGTG | TACCCGAGAGCTTGGCACCCA | MGEs:  Integron | integrase |
| *intI-1LC* | GGCATCCAAGCAGCAAG | AAGCAGACTTGACCTGA |  | integrase |
| *intI2* | TGCTTTTCCCACCCTTACC | GACGGCTACCCTCTGTTATCTC |  | integrase |
| *intI3* | GCCACCACTTGTTTGAGGA | GGATGTCTGTGCCTGCTTG |  | integrase |
| *IS613* | AGGTTCGGACTCAATGCAACA | TTCAGCACATACCGCCTTGAT | MGEs: Transposase | transposase |
| *tnpA-01* | CATCATCGGACGGACAGAATT | GTCGGAGATGTGGGTGTAGAAAGT |  | transposase |
| *tnpA-02* | GGGCGGGTCGATTGAAA | GTGGGCGGGATCTGCTT |  | transposase |
| *tnpA-03* | AATTGATGCGGACGGCTTAA | TCACCAAACTGTTTATGGAGTCGTT |  | transposase |
| *tnpA-04* | CCGATCACGGAAAGCTCAAG | GGCTCGCATGACTTCGAATC |  | transposase |
| *tnpA-05* | GCCGCACTGTCGATTTTTATC | GCGGGATCTGCCACTTCTT |  | transposase |
| *tnpA-07* | GAAACCGATGCTACAATATCCAATTT | CAGCACCGTTTGCAGTGTAAG |  | transposase |
| *Tp614* | GGAAATCAACGGCATCCAGTT | CATCCATGCGCTTTTGTCTCT |  | transposase |
| *carB* | GGAGTGAGGCTGACCGTAGAAG | ATCGGCGAAACGCACAAA | MLSB | efflux |
| *ereA* | CCTGTGGTACGGAGAATTCATGT | ACCGCATTCGCTTTGCTT |  | deactivate |
| *ereB* | GCTTTATTTCAGGAGGCGGAAT | TTTTAAATGCCACAGCACAGAATC |  | deactivate |
| *erm(34)* | GCGCGTTGACGACGATTT | TGGTCATACTCGACGGCTAGAAC |  | protection |
| *erm(35)* | TTGAAAACGATGTTGCATTAAGTCA | TCTATAATCACAACTAACCACTTGAACGT |  | protection |
| *erm(36)* | GGCGGACCGACTTGCAT | TCTGCGTTGACGACGGTTAC |  | protection |
| *ermA* | TTGAGAAGGGATTTGCGAAAAG | ATATCCATCTCCACCATTAATAGTAAACC |  | protection |
| *ermA/ermTR* | ACATTTTACCAAGGAACTTGTGGAA | GTGGCATGACATAAACCTTCATCA |  | protection |
| *ermB* | TAAAGGGCATTTAACGACGAAACT | TTTATACCTCTGTTTGTTAGGGAATTGAA |  | protection |
| *ermC* | TTTGAAATCGGCTCAGGAAAA | ATGGTCTATTTCAATGGCAGTTACG |  | protection |
| *ermF* | CAGCTTTGGTTGAACATTTACGAA | AAATTCCTAAAATCACAACCGACAA |  | protection |
| *ermJ/ermD* | GGACTCGGCAATGGTCAGAA | CCCCGAAACGCAATATAATGTT |  | protection |
| *ermK-01* | GTTTGATATTGGCATTGTCAGAGAAA | ACCATTGCCGAGTCCACTTT |  | protection |
| *ermK-02* | GAGCCGCAAGCCCCTTT | GTGTTTCATTTGACGCGGAGTAA |  | protection |
| *ermT-01* | GTTCACTAGCACTATTTTTAATGACAGAAGT | GAAGGGTGTCTTTTTAATACAATTAACGA |  | protection |
| *ermT-02* | GTAAAATCCCTAGAGAATACTTTCATCCA | TGAGTGATATTTTTGAAGGGTGTCTT |  | protection |
| *ermX* | GCTCAGTGGTCCCCATGGT | ATCCCCCCGTCAACGTTT |  | protection |
| *ermY* | TTGTCTTTGAAAGTGAAGCAACAGT | TAACGCTAGAGAACGATTTGTATTGAG |  | protection |
| *ImrA-01* | TCGACGTGACCGTAGTGAACA | CGTGACTACCCAGGTGAGTTGA |  | efflux |
| *lnuA-01* | TGACGCTCAACACACTCAAAAA | TTCATGCTTAAGTTCCATACGTGAA |  | deactivate |
| *lnuB-01* | TGAACATAATCCCCTCGTTTAAAGAT | TAATTGCCCTGTTTCATCGTAAATAA |  | deactivate |
| *lnuB-02* | AAAGGAGAAGGTGACCAATACTCTGA | GGAGCTACGTCAAACAACCAGTT |  | deactivate |
| *lnuC* | TGGTCAATATAACAGATGTAAACCAGATTT | CACCCCAGCCACCATCAA |  | deactivate |
| *matA/mel* | TAGTAGGCAAGCTCGGTGTTGA | CCTGTGCTATTTTAAGCCTTGTTTCT |  | efflux |
| *mdtA* | CCTAACGGGCGTGACTTCA | TTCACCTGTTTCAAGGGTCAAA |  | efflux |
| *mefA* | CCGTAGCATTGGAACAGCTTTT | AAACGGAGTATAAGAGTGCTGCAA |  | efflux |
| *mphA-01* | CTGACGCGCTCCGTGTT | GGTGGTGCATGGCGATCT |  | deactivate |
| *mphA-02* | TGATGACCCTGCCATCGA | TTCGCGAGCCCCTCTTC |  | deactivate |
| *mphB* | CGCAGCGCTTGATCTTGTAG | TTACTGCATCCATACGCTGCTT |  | deactivate |
| *mphC* | CGTTTGAAGTACCGAATTGGAAA | GCTGCGGGTTTGCCTGTA |  | deactivate |
| *msrA-01* | CTGCTAACACAAGTACGATTCCAAAT | TCAAGTAAAGTTGTCTTACCTACACCATT |  | efflux |
| *msrC-01* | TCAGACCGGATCGGTTGTC | CCTATTTTTTGGAGTCTTCTCTCTAATGTT |  | efflux |
| *oleC* | CCCGGAGTCGATGTTCGA | GCCGAAGACGTACACGAACAG |  | efflux |
| *pikR1* | TCGACATGCGTGACGAGATT | CCGCGAATTAGGCCAGAA |  | protection |
| *pikR2* | TCGTGGGCCAGGTGAAGA | TTCCCCTTGCCGGTGAA |  | protection |
| *vatB-01* | GGAAAAAGCAACTCCATCTCTTGA | TCCTGGCATAACAGTAACATTCTGA |  | deactivate |
| *vatB-02* | TTGGGAAAAAGCAACTCCATCT | CAATCCACACATCATTTCCAACA |  | deactivate |
| *vatC-01* | CGGAAATTGGGAACGATGTT | GCAATAATAGCCCCGTTTCCTA |  | deactivate |
| *vatC-02* | CGATGTTTGGATTGGACGAGAT | GCTGCAATAATAGCCCCGTTT |  | deactivate |
| *vatE-01* | GGTGCCATTATCGGAGCAAAT | TTGGATTGCCACCGACAAT |  | deactivate |
| *vatE-02* | GACCGTCCTACCAGGCGTAA | TTGGATTGCCACCGACAATT |  | deactivate |
| *vgaA-01* | CGAGTATTGTGGAAAGCAGCTAGTT | CCCGTACCGTTAGAGCCGATA |  | efflux |
| *vgaA-02* | GACGGGTATTGTGGAAAGCAA | TTTCCTGTACCATTAGATCCGATAATT |  | efflux |
| *vgb-01* | AGGGAGGGTATCCATGCAGAT | ACCAAATGCGCCCGTTT |  | deactivate |
| *vgbB-01* | CAGCCGGATTCTGGTCCTT | TACGATCTCCATTCAATTGGGTAAA |  | efflux |
| *vgbB-02* | ATACGAGCTGCCTAATAAAGGATCTT | TGTGAACCACAGGGCATTATCA |  | deactivate |
| *acrA-01* | CAACGATCGGACGGGTTTC | TGGCGATGCCACCGTACT | Non-specific | efflux |
| *acrA-02* | GGTCTATCACCCTACGCGCTATC | GCGCGCACGAACATACC |  | efflux |
| *acrA-03* | CAGACCCGCATCGCATATT | CGACAATTTCGCGCTCATG |  | efflux |
| *acrA-04* | TACTTTGCGCGCCATCTTC | CGTGCGCGAACGAACAT |  | efflux |
| *acrB-01* | AGTCGGTGTTCGCCGTTAAC | CAAGGAAACGAACGCAATACC |  | efflux |
| *acrR-01* | GCGCTGGAGACACGACAAC | GCCTTGCTGCGAGAACAAA |  | efflux |
| *acrR-02* | GATGATACCCCCTGCTGTGAGA | ACCAAACAAGAAGCGCAAGAA |  | efflux |
| *adeA* | CAGTTCGAGCGCCTATTTCTG | CGCCCTGACCGACCAAT |  | efflux |
| *acrA-05* | CGTGCGCGAACGAACA | ACTTTGCGCGCCATCTTC |  | efflux |
| *acrF* | GCGGCCAGGCACAAAA | TACGCTCTTCCCACGGTTTC |  | efflux |
| *ceoA* | ATCAACACGGACCAGGACAAG | GGAAAGTCCGCTCACGATGA |  | efflux |
| *cmeA* | GCAGCAAAGAAGAAGCACCAA | AGCAGGGTAAGTAAAACTAAGTGGTAAATCT |  | efflux |
| *cmr* | CGGCATCGTCAGTGGAATT | CGGTTCCGAAAAAGATGGAA |  | efflux |
| *emrD* | CTCAGCAGTATGGTGGTAAGCATT | ACCAGGCGCCGAAGAAC |  | efflux |
| *marR-01* | GCGGCGTACTGGTGAAGCTA | TGCCCTGGTCGTTGATGA |  | efflux |
| *mdetl1* | ATACAGCAGTGGATATTGGTTTAATTGT | TGCATAAGGTGAATGTTCCATGA |  | efflux |
| *mdtE/yhiU* | CGTCGGCGCACTCGTT | TCCAGACGTTGTACGGTAACCA |  | efflux |
| *mepA* | ATCGGTCGCTCTTCGTTCAC | ATAAATAGGATCGAGCTGCTGGAT |  | efflux |
| *mexA* | AGGACAACGCTATGCAACGAA | CCGGAAAGGGCCGAAAT |  | efflux |
| *mexD* | TTGCCACTGGCTTTCATGAG | CACTGCGGAGAACTGTCTGTAGA |  | efflux |
| *mexE* | GGTCAGCACCGACAAGGTCTAC | AGCTCGACGTACTTGAGGAACAC |  | efflux |
| *mexF* | CCGCGAGAAGGCCAAGA | TTGAGTTCGGCGGTGATGA |  | efflux |
| *mtrC-01* | GGACGGGAAGATGGTCCAA | CGTAGCGTTCCGGTTCGAT |  | efflux |
| *mtrC-02* | CGGAGTCCATCGACCATTTG | ATCGTCGGCAAGGAGAATCA |  | efflux |
| *mtrD-02* | GGTCGGCACGCTCTTGTC | TGAAGAATTTGCGCACCACTAC |  | efflux |
| *mtrD-03* | CCGCCAAGCCGATATAGACA | GGCCGGGTTGCCAAA |  | efflux |
| *oprD* | ATGAAGTGGAGCGCCATTG | GGCCACGGCGAACTGA |  | efflux |
| *oprJ* | ACGAGAGTGGCGTCGACAA | AAGGCGATCTCGTTGAGGAA |  | efflux |
| *pmrA* | TTTGCAGGTTTTGTTCCTAATGC | GCAGAGCCTGATTTCTCCTTTG |  | efflux |
| *putative multidrug* | AATTTTGCCGATTATTGCTGAAA | GATTGTCATCATTCGTTTATCACCAA |  | efflux |
| *qac* | CAATAATAACCGAAATAATAGGGACAAGTT | AATAAGTGTTCCTAGTGTTGGCCATAG |  | efflux |
| *qacA* | TGGCAATAGGAGCTATGGTGTTT | AAGGTAACACTATTTTCGGTCCAAATC |  | efflux |
| *qacA/qacB* | TTTAGGCAGCCTCGCTTCA | CCGAATCCAAATAAAACCCAATAA |  | efflux |
| *qacEdelta1-01* | TCGCAACATCCGCATTAAAA | ATGGATTTCAGAACCAGAGAAAGAAA |  | efflux |
| *qacEdelta1-02* | CCCCTTCCGCCGTTGT | CGACCAGACTGCATAAGCAACA |  | efflux |
| *qacH-01* | GTGGCAGCTATCGCTTGGAT | CCAACGAACGCCCACAA |  | efflux |
| *qacH-02* | CATCGTGCTTGTGGCAGCTA | TGAACGCCCAGAAGTCTAGTTTT |  | efflux |
| *rarD-02* | TGACGCATCGCGTGATCT | AAATTTTCTGTGGCGTCTGAATC |  | efflux |
| *sdeB* | CACTACCGCTTCCGCACTTAA | TGAAAAAACGGGAAAAGTCCAT |  | efflux |
| *tolC-01* | GGCCGAGAACCTGATGCA | AGACTTACGCAATTCCGGGTTA |  | efflux |
| *tolC-02* | CAGGCAGAGAACCTGATGCA | CGCAATTCCGGGTTGCT |  | efflux |
| *tolC-03* | GCCAGGCAGAGAACCTGATG | CGCAATTCCGGGTTGCT |  | efflux |
| *ttgA* | ACGCCAATGCCAAACGATT | GTCACGGCGCAGCTTGA |  | efflux |
| *ttgB* | TCGCCCTGGATGTACACCTT | ACCATTGCCGACATCAACAAC |  | efflux |
| *yceE/mdtG-01* | TGGCACAAAATATCTGGCAGTT | TTGTGTGGCGATAAGAGCATTAG |  | efflux |
| *yceE/mdtG-02* | TTATCTGTTTTCTGCTCACCTTCTTTT | GCGTGGTGACAAACAGGCTTA |  | efflux |
| *yceL/mdtH-01* | TCGGGATGGTGGGCAAT | CGATAACCGAGCCGATGTAGA |  | efflux |
| *yceL/mdtH-02* | CGCGTGAAACCTTAAGTGCTT | AGACGGCTAAACCCCATATAGCT |  | efflux |
| *yceL/mdtH-03* | CTGCCGTTAAATGGATGTATGC | ACTCCAGCGGGCGATAGG |  | efflux |
| *yidY/mdtL-01* | GCAGTTGCATATCGCCTTCTC | CTTCCCGGCAAACAGCAT |  | efflux |
| *yidY/mdtL-02* | TGCTGATCGGGATTCTGATTG | CAGGCGCGACGAACATAAT |  | efflux |
| *fabK* | TTTCAGCTCAGCACTTTGGTCAT | AAGGCATCTTTTTCAGCCAGTTC | Other | deactivate |
| *imiR* | CCGGACTAGAGCTTCATGTAAGC | CCCACGCGGTACTCTTGTAAA |  | unknown |
| *nisB* | GGGAGAGTTGCCGATGTTGTA | AGCCACTCGTTAAAGGGCAAT |  | unknown |
| *speA* | GCAAGAGGTATTTGCTCAACAAGA | CAGGGTCACCCTCATAAAGAAAA |  | unknown |
| *bacA-01* | CGGCTTCGTGACCTCGTT | ACAATGCGATACCAGGCAAAT |  | deactivate |
| *bacA-02* | TTCCACGACACGATTAAGTCATTG | CGGCTCTTTCGGCTTCAG |  | deactivate |
| *fosB* | TCACTGTAACTAATGAAGCATTAGACCAT | CCATCTGGATCTGTAAAGTAAAGAGATC |  | deactivate |
| *fosX* | GATTAAGCCATATCACTTTAATTGTGAAAG | TCTCCTTCCATAATGCAAATCCA |  | deactivate |
| *nimE* | TGCGCCAAGATAGGGCATA | GTCGTGAATTCGGCAGGTTTA |  | unknown |
| *pncA* | GCAATCGAGGCGGTGTTC | TTGCCGCAGCCAATTCA |  | unknown |
| *sat4* | GAATGGGCAAAGCATAAAAACTTG | CCGATTTTGAAACCACAATTATGATA |  | deactivate |
| *dfrA1* | GGAATGGCCCTGATATTCCA | AGTCTTGCGTCCAACCAACAG | Sulfonamide | deactivate |
| *dfrA12* | CCTCTACCGAACCGTCACACA | GCGACAGCGTTGAAACAACTAC |  | deactivate |
| *folA* | CGAGCAGTTCCTGCCAAAG | CCCAGTCATCCGGTTCATAATC |  | deactivate |
| *sul1* | CAGCGCTATGCGCTCAAG | ATCCCGCTGCGCTGAGT |  | protection |
| *sul2* | TCATCTGCCAAACTCGTCGTTA | GTCAAAGAACGCCGCAATGT |  | protection |
| *sulA/folP-01* | CAGGCTCGTAAATTGATAGCAGAAG | CTTTCCTTGCGAATCGCTTT |  | protection |
| *sulA/folP-03* | CACGGCTTCGGCTCATGT | TGCCATCCTGTGACTAGCTACGT |  | protection |
| *tet(32)* | CCATTACTTCGGACAACGGTAGA | CAATCTCTGTGAGGGCATTTAACA | Tetracycline | protection |
| *tet(34)* | CTTAGCGCAAACAGCAATCAGT | CGGTGATACAGCGCGTAAACT |  | unknown |
| *tet(35)* | ACCCCATGACGTACCTGTAGAGA | CAACCCACACTGGCTACCAGTT |  | unknown |
| *tet(36)* | AGAATACTCAGCAGAGGTCAGTTCCT | TGGTAGGTCGATAACCCGAAAAT |  | protection |
| *tet(36)* | TGCAGGAAAGACCTCCATTACAG | CTTTGTCCACACTTCCACGTACTATG |  | protection |
| *tet(37)* | GAGAACGTTGAAAAGGTGGTGAA | AACCAAGCCTGGATCAGTCTCA |  | unknown |
| *tetA-01* | GCTGTTTGTTCTGCCGGAAA | GGTTAAGTTCCTTGAACGCAAACT |  | efflux |
| *tetA-02* | CTCACCAGCCTGACCTCGAT | CACGTTGTTATAGAAGCCGCATAG |  | efflux |
| *tetB-01* | AGTGCGCTTTGGATGCTGTA | AGCCCCAGTAGCTCCTGTGA |  | efflux |
| *tetB-02* | GCCCAGTGCTGTTGTTGTCAT | TGAAAGCAAACGGCCTAAATACA |  | efflux |
| *tetC-01* | CATATCGCAATACATGCGAAAAA | AAAGCCGCGGTAAATAGCAA |  | efflux |
| *tetC-02* | ACTGGTAAGGTAAACGCCATTGTC | ATGCATAAACCAGCCATTGAGTAAG |  | efflux |
| *tetD-01* | TGCCGCGTTTGATTACACA | CACCAGTGATCCCGGAGATAA |  | efflux |
| *tetD-02* | TGTCATCGCGCTGGTGATT | CATCCGCTTCCGGGAGAT |  | efflux |
| *tetE* | TTGGCGCTGTATGCAATGAT | CGACGACCTATGCGATCTGA |  | efflux |
| *tetG-01* | TCAACCATTGCCGATTCGA | TGGCCCGGCAATCATG |  | efflux |
| *tetG-02* | CATCAGCGCCGGTCTTATG | CCCCATGTAGCCGAACCA |  | efflux |
| *tetH* | TTTGGGTCATCTTACCAGCATTAA | TTGCGCATTATCATCGACAGA |  | efflux |
| *tetJ* | GGGTGCCGCATTAGATTACCT | TCGTCCAATGTAGAGCATCCATA |  | efflux |
| *tetK* | CAGCAGTCATTGGAAAATTATCTGATTATA | CCTTGTACTAACCTACCAAAAATCAAAATA |  | efflux |
| *tetL-01* | AGCCCGATTTATTCAAGGAATTG | CAAATGCTTTCCCCCTGTTCT |  | efflux |
| *tetL-02* | ATGGTTGTAGTTGCGCGCTATAT | ATCGCTGGACCGACTCCTT |  | efflux |
| *tetM-01* | CATCATAGACACGCCAGGACATAT | CGCCATCTTTTGCAGAAATCA |  | protection |
| *tetM-02* | TAATATTGGAGTTTTAGCTCATGTTGATG | CCTCTCTGACGTTCTAAAAGCGTATTAT |  | protection |
| *tetO-01* | ATGTGGATACTACAACGCATGAGATT | TGCCTCCACATGATATTTTTCCT |  | protection |
| *tetW-01* | ATGAACATTCCCACCGTTATCTTT | ATATCGGCGGAGAGCTTATCC |  | protection |
| *tetPA* | AGTTGCAGATGTGTATAGTCGTAAACTATCTATT | TGCTACAAGTACGAAAACAAAACTAGAA |  | efflux |
| *tetPB-01* | ACACCTGGACACGCTGATTTT | ACCGTCTAGAACGCGGAATG |  | protection |
| *tetPB-02* | TGATACACCTGGACACGCTGAT | CGTCCAAAACGCGGAATG |  | protection |
| *tetPB-03* | TGGGCGACAGTAGGCTTAGAA | TGACCCTACTGAAACATTAGAAATATACCT |  | protection |
| *tetPB-05* | CTGAAGTGGAGCGATCATTCC | CCCTCAACGGCAGAAATAACTAA |  | protection |
| *tetQ* | CGCCTCAGAAGTAAGTTCATACACTAAG | TCGTTCATGCGGATATTATCAGAAT |  | protection |
| *tetR-02* | CGCGATAGACGCCTTCGA | TCCTGACAACGAGCCTCCTT |  | efflux |
| *tetR-03* | CGCGATGGAGCAAAAGTACAT | AGTGAAAAACCTTGTTGGCATAAAA |  | efflux |
| *tetS* | TTAAGGACAAACTTTCTGACGACATC | TGTCTCCCATTGTTCTGGTTCA |  | protection |
| *tetT* | CCATATAGAGGTTCCACCAAATCC | TGACCCTATTGGTAGTGGTTCTATTG |  | protection |
| *tetU-01* | GTGGCAAAGCAACGGATTG | TGCGGGCTTGCAAAACTATC |  | unknown |
| *tetV* | GCGGGAACGACGATGTATATC | CCGCTATCTCACGACCATGAT |  | efflux |
| *tetX* | AAATTTGTTACCGACACGGAAGTT | CATAGCTGAAAAAATCCAGGACAGTT |  | unknown |
| *vanA* | AAAAGGCTCTGAAAACGCAGTTAT | CGGCCGTTATCTTGTAAAAACAT | Vancomycin | protection |
| *vanB-01* | TTGTCGGCGAAGTGGATCA | AGCCTTTTTCCGGCTCGTT |  | protection |
| *vanC-01* | ACAGGGATTGGCTATGAACCAT | TGACTGGCGATGATTTGACTATG |  | protection |
| *vanC-02* | CCTGCCACAATCGATCGTT | CGGCTTCATTCGGCTTGATA |  | protection |
| *vanC-03* | AAATCAATACTATGCCGGGCTTT | CCGACCGCTGCCATCA |  | protection |
| *vanC1* | AGGCGATAGCGGGTATTGAA | CAATCGTCAATTGCTCATTTCC |  | protection |
| *vanC2/vanC3* | TTTGACTGTCGGTGCTTGTGA | TCAATCGTTTCAGGCAATGG |  | protection |
| *vanG* | ATTTGAATTGGCAGGTATACAGGTTA | TGATTTGTCTTTGTCCATACATAATGC |  | protection |
| *vanHB* | GAGGTTTCCGAGGCGACAA | CTCTCGGCGGCAGTCGTAT |  | protection |
| *vanHD* | GTGGCCGATTATACCGTCATG | CGCAGGTCATTCAGGCAAT |  | protection |
| *vanRA-01* | CCCTTACTCCCACCGAGTTTT | TTCGTCGCCCCATATCTCAT |  | protection |
| *vanRA-02* | CCACTCCGGCCTTGTCATT | GCTAACCACATTCCCCTTGTTTT |  | protection |
| *vanRB* | GCCCTGTCGGATGACGAA | TTACATAGTCGTCTGCCTCTGCAT |  | protection |
| *vanRC* | TGCGGGAAAAACTGAACGA | CCCCCCATACGGTTTTGATTA |  | protection |
| *vanRC4* | AGTGCTTTGGCTTATCTCGAAAA | TCCGGCAGCATCACATCTAA |  | protection |
| *vanRD* | TTATAATGGCAAGGATGCACTAAAGT | CGTCTACATCCGGAAGCATGA |  | protection |
| *vanSA* | CGCGTCATGCTTTCAAAATTC | TCCGCAGAAAGCTCAATTTGTT |  | protection |
| *vanSB* | GCGCGGCAAATGACAAC | TTTGCCATTTTATTCGCACTGT |  | protection |
| *vanSC-01* | ATCAACTGCGGGAGAAAAGTCT | TCCGCTGTTCCGCTTCTT |  | protection |
| *vanSC-02* | GCCATCAGCGAGTCTGATGA | CAGCTGGGATCGTTTTTCCTT |  | protection |
| *vanTC-01* | CACACGCATTTTTTCCCATCTAG | CAGCCAACAGATCATCAAAACAA |  | protection |
| *vanTC-02* | ACAGTTGCCGCTGGTGAAG | CGTGGCTGGTCGATCAAAA |  | protection |
| *vanTE* | GTGGTGCCAAGGAAGTTGCT | CGTAGCCACCGCAAAAAAAT |  | protection |
| *vanTG* | CGTGTAGCCGTTCCGTTCTT | CGGCATTACAGGTATATCTGGAAA |  | protection |
| *vanWB* | CGGACAAAGATACCCCCTATAAAG | AAATAGTAAATTGCTCATCTGGCACAT |  | protection |
| *vanWG* | ACATTTTCATTTTGGCAGCTTGTAC | CCGCCATAAGAGCCTACAATCT |  | protection |
| *vanXA* | CGCTAAATATGCCACTTGGGATA | TCAAAAGCGATTCAGCCAACT |  | protection |
| *vanXB* | AGGCACAAAATCGAAGATGCTT | GGGTATGGCTCATCAATCAACTT |  | protection |
| *vanXD* | TAAACCGTGTTATGGGAACGAA | GCGATAGCCGTCCCATAAGA |  | protection |
| *vanYB* | GGCTAAAGCGGAAGCAGAAA | GATATCCACAGCAAGACCAAGCT |  | protection |
| *vanYD-01* | AAGGCGATACCCTGACTGTCA | ATTGCCGGACGGAAGCA |  | protection |
| *vanYD-02* | CAAACGGAAGAGAGGTCACTTACA | CGGACGGTAATAGGGACTGTTC |  | protection |

## References

1. Ott A, O’Donnell G, Tran NH, Haniffah MRBM, Su J-Q, Zealand AM, et al. Developing surrogate markers for predicting antibiotic resistance “hot spots” in rivers where limited data are available. Environ Sci Technol. 2021;55:7466–78.

2. Vandeputte D, Kathagen G, D’Hoe K, Vieira-Silva S, Valles-Colomer M, Sabino J, et al. Quantitative microbiome profiling links gut community variation to microbial load. Nature. 2017;551:507–11. doi:10.1038/nature24460.

3. Alberdi A, Gilbert MTP. A guide to the application of Hill numbers to DNA-based diversity analyses. Mol Ecol Resour. 2019;19:804–17.

4. Alberdi A, Gilbert MTP. hilldiv: an R package for the integral analysis of diversity based on Hill numbers. bioRxiv. 2019;1 February:545665. doi:10.1101/545665.

5. Zhu YG, Johnson TA, Su JQ, Qiao M, Guo GX, Stedtfeld RD, et al. Diverse and abundant antibiotic resistance genes in Chinese swine farms. Proc Natl Acad Sci U S A. 2013;110:3435–40.

6. Zhu YG, Zhao Y, Li B, Huang CL, Zhang SY, Yu S, et al. Continental-scale pollution of estuaries with antibiotic resistance genes. Nat Microbiol. 2017;2 January.
